# Selective synthesis of thioethers in the presence of a transition-metal-free solid Lewis acid

**DOI:** 10.3762/bjoc.12.259

**Published:** 2016-12-06

**Authors:** Federica Santoro, Matteo Mariani, Federica Zaccheria, Rinaldo Psaro, Nicoletta Ravasio

**Affiliations:** 1 CNR ISTM, via C. Golgi 19, 20133 Milano, Italy

**Keywords:** no solvent, S-alkylation, solid acids, thioethers, transition-metal-free

## Abstract

The synthesis of thioethers starting from alcohols and thiols in the presence of amorphous solid acid catalysts is reported. A silica alumina catalyst with a very low content in alumina gave excellent results in terms of both activity and selectivity also under solvent-free conditions. The reaction rate follows the electron density of the carbinol atom in the substrate alcohol and yields up to 99% and can be obtained for a wide range of substrates under mild reaction conditions.

## Introduction

The need for more sustainable processes in the fine chemical industry is growing continuously. An optimal use of resources, both energy and starting materials, and a consequent waste reduction can be recognized as important factors for environmental protection. In this context organic synthesis over heterogeneous catalysts instead of homogeneous ones or without employing any organic solvents is of paramount interest [1–2]. In particular, metal-free coupling reactions are a very important field of research as traditional coupling methods, although they proved effective in industrial applications, generate harmful metal waste and many byproducts [3].

Thioethers are important building blocks for the synthesis of antibacterial and antifungal agents [4–5] and as antioxidants in polymers [6]. They are typically synthesized through the condensation of a thiol with organic halides under strong basic conditions [7–9], but due to the high toxicity of alkyl halides the introduction of new methods of access to this kind of materials is desirable. The ideal reaction from the green chemistry point of view would be the direct substitution of alcohols (that are also available at low cost) with thiols. In this case the only byproduct will be water. However, due to the lack of a good leaving group the use of an acid catalyst is mandatory. Both Brønsted and Lewis acids can be used. The former ones, such as free or polymer bound *p*-toluenesulfonic acid, promote the formation of significant amounts of by-products and can give yields in the range of 80% only for propargylic, allylic or benzylic alcohols [10–14]. As far as Lewis acids are concerned ZrCl_4_ [15] dispersed on silica is active in promoting the substitution of adamantanol, cinnamyl and benzyl alcohols with thiols whereupon significative amounts of Zr salt are required; whereas Wu and Han have shown that Ga(OTf)_3_ is an effective catalyst for the substitution of a wide range of benzylic and allylic alcohols with phosphorothioic acid and with a wide range of alcohols with various sulfur nucleophiles in an effective way [16]. On the contrary SmCl_3_ promotes the formation of thioethers only from 2-cyclohexen-1-ol and geraniol with thiophenol [17] while the use of cationic diruthenium complexes is limited to the displacement of propargyl alcohols with thiols [18].

Heterogeneous systems are very rare. Corma and Sabater used a heterogeneous system based on palladium on magnesium oxide under borrowing hydrogen conditions [19]. The reaction has to be carried out at 180 °C under N_2_ in trifluorotoluene with a maximum yield of 83% and it can be used only for primary benzylic alcohols. On the contrary Ni nanoparticles act as chemoselective catalysts at room temperature [20]. 1,3,5-Triazo-2,4,6-triphosphorine-2,2,4,4,6,6-hexachloride (TAPC) allows the efficient preparation of thioethers from different thiols and benzylic alcohols under solvent-free conditions in excellent yields [21].

We already reported on the use of amorphous solid acid catalysts in organic synthesis. These solids are formed by dispersing a small amount of an inorganic oxide with Lewis acid nature onto the surface of silica [22]. In particular we found that 1-(4-methoxyphenyl)ethanol promptly reacts with 2-PrOH at 80 °C to give the asymmetric ether in 92% yield in the presence of a silica alumina mixed oxide [23]. This prompted us to investigate the reactivity of aromatic alcohols with thiols under the same conditions. Here we wish to report that excellent yields can be obtained in the presence of an amorphous solid catalyst under solvent-free conditions.

## Results and Discussion

In order to test our hypothesis we carried out the reaction of 1-(4-methoxyphenyl)ethanol (**1a**) and benzyl mercaptan (**2a**) in the presence of different solid acids, namely 13% Al_2_O_3_ on silica (SiAl 13), 4.7% ZrO_2_ on silica (SiZr 4.7), 2.3% TiO_2_ on silica (SiTi 2.3) and 0.6% Al_2_O_3_ on silica (SiAl 0.6) whose textural properties are summed up in Supporting Information File 1, Table S1. Results are reported in Table 1.

**Table 1:** Thioethers synthesis in solvent with different catalysts^a^.

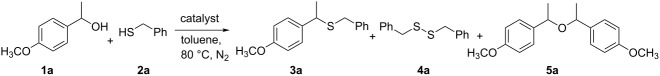								

Entry	Catalyst	N_OH_/nm^2^	*t* (h)	Conv. (%)	**3a** (%)^b^	**4a** (%)^b^	**5a** (%)^b^	Dehydr. (%)^b,c^

1	No cat.	–	2	6.3	25.0	34.4	18.7	21.9

2	SiAl 13	11.5	0.5	15.8	66.9	–	25.7	7.4
			2	67.6	94.3	–	3.0	2.7

3	SiZr 4.7	7.35	0.5	47.4	93.5	–	6.5	–
			2	86.1	98.7	–	0.8	0.5

4	SiTi 2.3	4.85	0.5	71.2	92.2	0.1	3.0	4.7
			2	93.4	95.5	0.1	0.1	4.3

5	SiAl 0.6	3.55	0.5	93.4	98.8	–	1.0	0.2
			2	>99	99.9	–	–	0.1

^a^Reaction conditions: cat. = 100 mg, cat./ROH = 1:1 (w/w), ROH/RSH = 1:1 (mol/mol), toluene (8 mL), N_2_ (1 atm), 80 °C (oil bath temp.), stirring (1000 rpm); reaction mixtures were analyzed by GC–MS (5% phenylmethyl polysiloxane capillary column, length 30 m, injection *T* = 60 °C), and by ^1^H NMR and ^13^C NMR spectroscopy; conversion was calculated with respect to the thiol. ^b^percentage composition of reaction products. ^c^Corresponding substituted styrene derived from alcohol dehydration.

It is worth underlining that under these experimental conditions the reaction does not proceed in the absence of a catalyst (Table 1, entry 1). Only a very low conversion was obtained with statistical product distribution. On the contrary all the four solid catalysts were found to be active in this reaction (Table 1, entries 2–5). This is in agreement with the acidic character of these materials, often shown by our group. In particular we reported on the relevant activity and robustness of SiZr 4.7 in the esterification of fatty acids with methanol [24] or with polyols [25–26].

However, significant differences were found among the four solids. Both activity and selectivity depend on the hydrophilic character of the solid, here represented by the number of hydroxy groups per surface area unit (N_OH_/nm^2)^. The lower this parameter the higher are both reaction rate and selectivity.

This is particularly evident from the results obtained after 0.5 h reaction. When the surface hydroxy group number is higher as in SiAl 13, not only the activity but also the selectivity is very low (Table 1, entry 2). This may well be due to the hydrophilic surface preferentially attracting the alcohol molecules. Thus, at the beginning we can observe the formation of the symmetrical ether formed through reaction of two alcohol molecules, beside the desired product. For longer reaction times the selectivity increases due to conversion of the ether initially formed as it is suggested from data summed up in Table 1.

**Figure 1:**
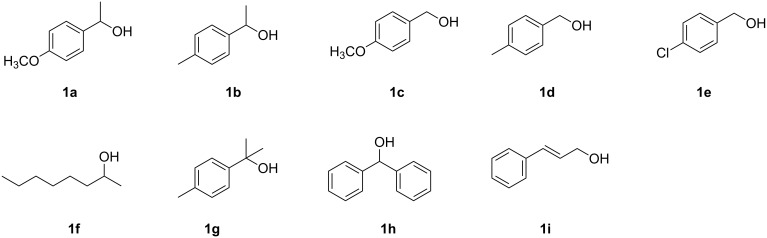
Overview of the structures of the alcohols **1a–i** used in the present work.

**Figure 2:**
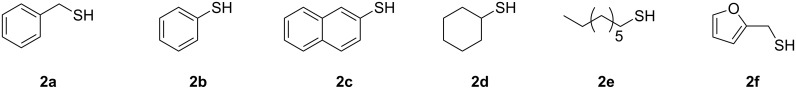
Structures of thiols **2a–f** used in the present work.

**Figure 3:**
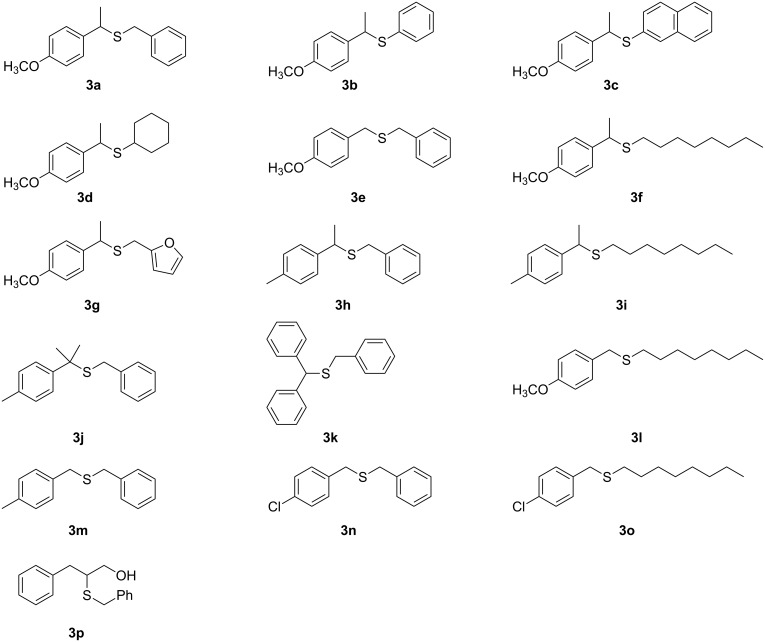
Structures of thioethers **3a–p** synthesized.

In particular a catalyst with a very low loading of alumina on silica and a very low number of surface hydroxy groups gave quantitatively the desired product in 2 hours (Table 1, entry 5). The activity of this solid has to be ascribed to the presence of well dispersed Lewis acid sites on the surface, as put in evidence from the FTIR spectrum of adsorbed pyridine where the band due to Lewis acid sites is detectable (1456 cm^−1^). This excellent performance prompted us to investigate the substrate scope of this reaction in the presence of SiAl 0.6. Figure 1 and Figure 2 report alcohols and thiols used as reagents while Figure 3 lists the products obtained.

A preliminary study on the substrate scope of this reaction summed up in Supporting Information File 1, Table S2 showed that only benzylic alcohols can be transformed under these conditions, particularly secondary ones, whereas secondary cyclic and acyclic aliphatic alcohols were found to be totally unreactive. As far as the thiol is concerned both aromatic and cyclic thiols gave excellent results.

The attempt to carry out the reaction in a solvent-free mode gave surprising results. The reaction was slower but selectivity was still very high. Results are reported in Table 2. Aromatic and aliphatic thiols gave excellent results in the reaction with **1a** whereas the structure of the alcohol had a more significant impact on the reactivity (Table 2, entries 1–7). Thus, **1b** was much less reactive than **1a** and to reach a 94% yield the temperature had to be raised to 110 °C. Moreover the reaction at the beginning gave almost equimolecular amounts of the thioether and the ether, although during time the ether converted into the desired product (Table 2, entry 9).

**Table 2:** Synthesis of thioethers from different alcohols and thiols promoted by SiAl 0.6 without solvent^a^.

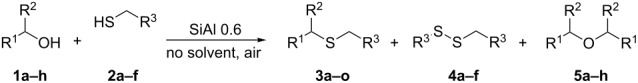								

Entry	**1a–h**	**2a–f**	*T* (°C)^b^	*t* (h)	Conv. (%)	**3a–o**^c^ (%)	**4a–f**^c^(%)	**5a–h**^c^ (%)

1	**1a**	**2a**				**3a**	**4a**	**5a**
			60	0.5	60	75.0	5.5	12.3
				1	97	94.8	1.4	3.8
				2	>99	99.0	0.6	0.4

2	**1a**	**2b**				**3b**	**4b**	**5a**
			60	0.5	74	80.2	3.5	12.8
				1	90	88.3	1.7	9.9
				2	98	98.9	1.1	–

3	**1a**	**2c**				**3c**	**4c**	**5a**
			60	0.5	57	63.5	20.7	13.9
				2	82	76.3	4.8	15.5
				5	>99	99.4	0.6	–

4	**1a**	**2c**				**3c**	**4c**	**5a**
			90	0.5	>99	98.7	1.3	–

5	**1a**	**2d**				**3d**	**4d**	**5a**
			60	0.5	56	56.6	1.4	32.1
				3	99	99.6	0.4	–

6	**1a**	**2e**				**3f**	**4e**	**5a**
			60	2	82	85.1	1.7	13.2
				4.5	>99	98.9	1.1	–

7	**1a**	**2f**				**3g**	**4f**	**5a**
			60	1	85	68.4	–	31.6
				3	99	86.9	–	1.5

8	**1b**	**2a**				**3h**	**4a**	**5b**
			60	2	3	29.4	32.9	37.8
				20	16	46.1	48.3	5.6

9	**1b**	**2a**				**3h**	**4a**	**5b**
			110	0.5	74	55.0	–	45.0
				6	99	74.4	1.0	24.6
				12	>99	86.8	1.4	12.1
				20	>99	95.2	1.5	3.3

10	**1b**	**2e**				**3i**	**4e**	**5b**
			110	0.5	89	72.2	2.1	25.7
				2	>99	93.1	2.2	4.7

11	**1g**	**2a**				**3j**	**4a**	**5g**
			90	0.5	>99	94.4	–	0.9

12	**1h**	**2a**				**3k**	**4a**	**5h**
			60	0.5	14	89.5	10.5	–
				2	40	87.2	3.5	9.3
				20	95	91.3	0.7	8.0

13	**1c**	**2a**				**3e**	**4a**	**5c**
			90	6	98	92.8	1.0	6.2

14	**1c**	**2a**				**3e**	**4a**	**5c**
			110	0.5	99	93.8	1.2	5.0
				1	>99	97.7	1.1	1.2

15	**1c**	**2e**				**3l**	**4e**	**5c**
			90	0.5	57	41.9	1.8	52.8
				4	96	86.7	1.5	8.5

16	**1c**	**2e**				**3l**	**4e**	**5c**
			110	0.5	87	78	1	20
				1	97	91.9	1.2	5.6

17	**1d**	**2a**				**3m**	**4a**	**5d**
			90	6	2	–	>99	–

18	**1d**	**2a**				**3m**	**4a**	**5d**
			110	6	9	20.2	18.0	46.1

19	**1e**	**2a**				**3n**	**4a**	**5e**
			110	20	15	3.6	–	61.8

20	**1e**	**2e**				**3o**	**4e**	**5e**
			110	12	52	4.8	50.2	–

21	**1f**	**2e**				**3w**	**4e**	**5f**
			110	20	8	79.9	20.1	–

^a^Reaction conditions: cat. = 10 mg cat./ROH = 1:10 (w/w), ROH/RSH = 1:1 (mol/mol), no solvent, air, magnetic stirring (1000 rpm); reaction mixtures were analysed by GC–MS (5% phenylmethyl polysiloxane capillary column length 30 m, injection *T* = 60 °C), and by ^1^H NMR and ^13^C NMR spectroscopy; conversion was calculated with respect to the thiol. ^b^Oil bath temperature. ^c^Percentage composition of reaction products.

**Figure 4:**
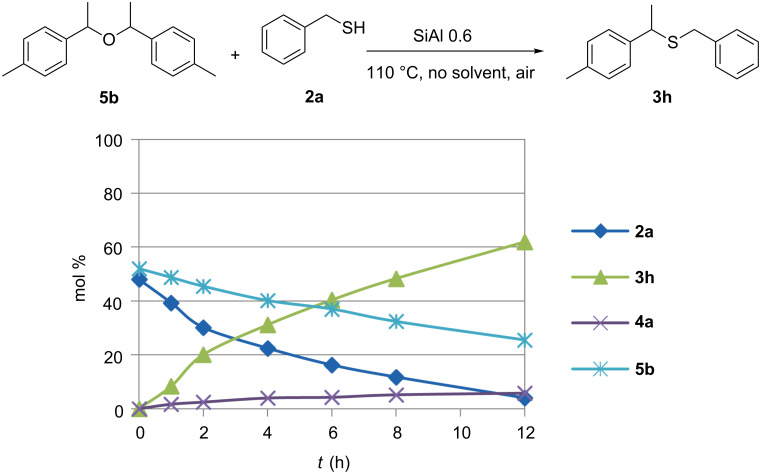
Product distribution during reaction of **5b** and **2a** over a solid acid catalyst.

The higher reactivity of the substrate bearing the more electron donating group suggests that the reaction takes place through a nucleophilic substitution mechanism: the higher the electronic density on the carbinol C atom, the higher the reaction rate. When the electronic density is somewhat lower, competitive formation of the ether occurs but subsequent nucleophilic addition of the thiol to the ether restores a very high selectivity. A test carried out by reacting presynthetized ether 4,4'-(oxybis(ethane-1,1-diyl))bis(methylbenzene) (**5b**) with benzyl mercaptan (**2a**) showed indeed that the thioether is formed easily starting from these two molecules under the reaction conditions reported (Figure 4).

**Figure 5:**
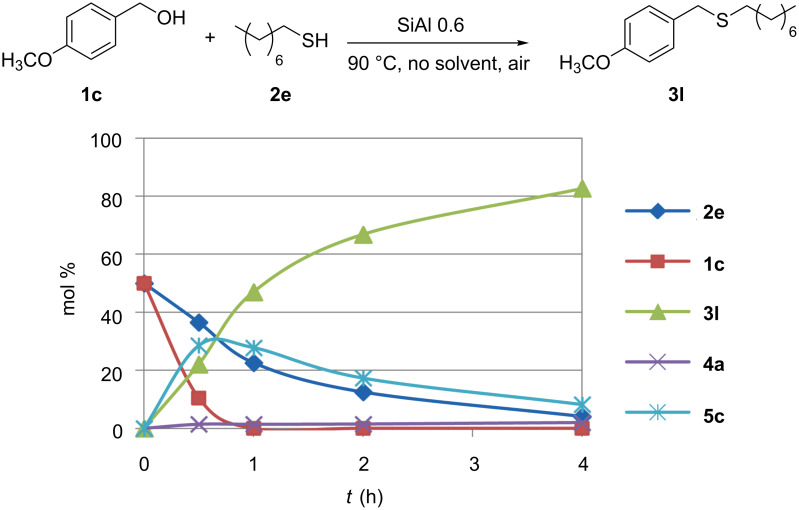
Product distribution during reaction of **1c** and **2e**.

The dependence of the reaction rate on the stability of the intermediate carbocation is even more evident in the series of primary benzylic alcohols. Among them only the *p*-methoxy-substituted compound **1c** shows high activity. In particular, with thiol **2e** some ether was formed that during time is converted into the product (Table 2, entry 15 and Figure 5).

On the other hand highly hindered alcohol **1g** with a tertiary carbinol atom gave a very fast and selective reaction (Table 2, entry 11).

**Scheme 1:**
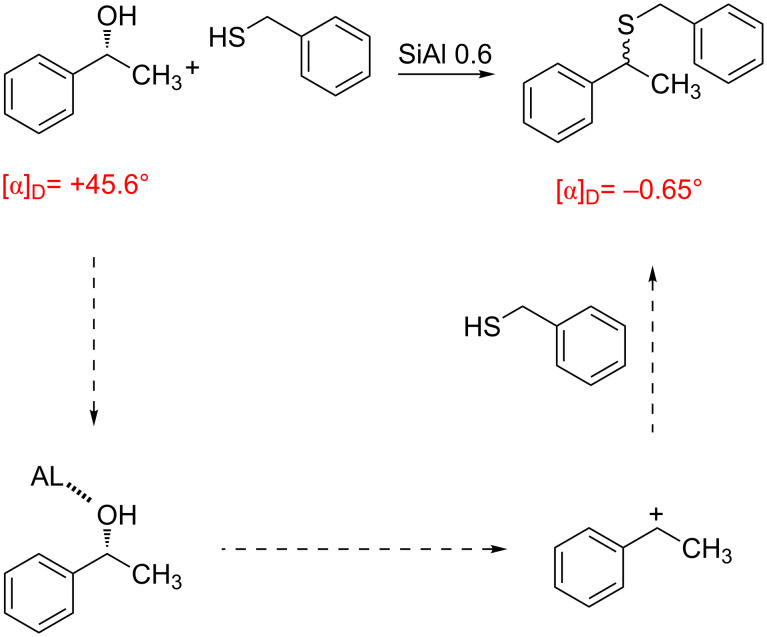
Racemization of (*R*)-1-phenylethanol during the reaction with benzylmercaptan (**2a**) in the presence of SiAl 0.6.

Thus we can conclude that the reaction rate follows the carbocation stability according to an SN1 mechanism. To confirm this hypothesis the reaction of optically active (*R*)-1-phenylethanol gave a completely racemic compound (Scheme 1).

**Scheme 2:**
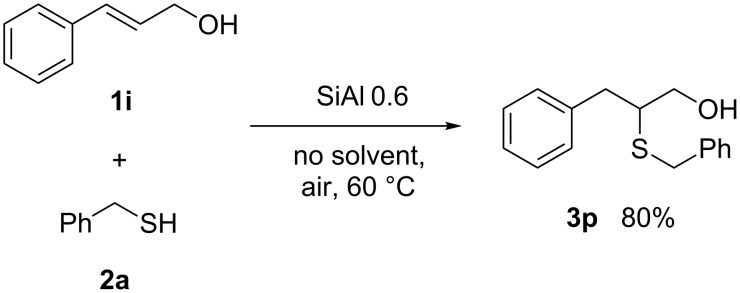
Reaction of cinnamyl alcohol **1i** and benzylmercaptan (**2a**).

Unfortunately allylic alcohols gave unreproducible results. However, in the case of cinnamyl alcohol (**1i**) we could obtain a fairly good selectivity to the product of the thiol–ene reaction **3p** (Scheme 2).

The protocol here described is very advantageous from the point of view of Green Chemistry. The direct substitution of hydroxy groups in alcohols is very difficult and generally they have to be converted into better leaving groups, typically halides, before reaction with nucleophiles. This process generates salt waste limiting industrial application. Moreover these compounds are generally toxic. The development of catalytic alternative methods for this reaction is highly sought after. However, the catalytic transformation of thiols is much less developed than that of amines and alcohols. This is due to the fact that transition metal ions are strongly thiophilics, therefore most of metallic catalysts are poisoned by the presence of sulfur compounds. Lanthanide complexes such as Yb(OTf)_3_ are an exception [27] but only allylic and propargylic alcohols react under the reported conditions. The alkylation of thiols has also been carried out under flow conditions in the presence of a heterogeneous base in a packed bed reactor but also in this case alkyl halides were used as electrophiles [28].

**Figure 6:**
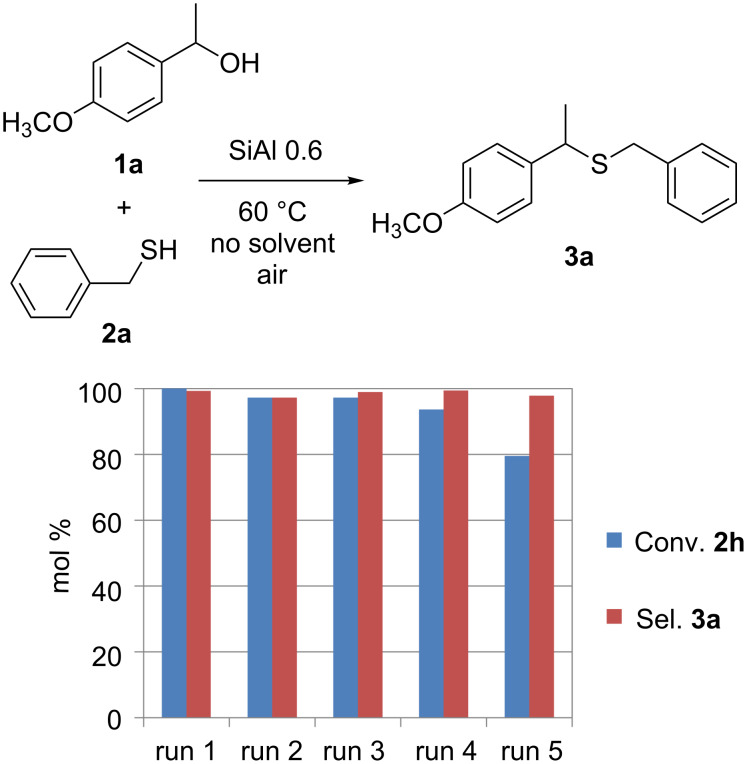
Recyclability test of SiAl 0.6 catalyst in the reaction of **1a** and **2a**.

The reaction we are presenting here takes place in the presence of a solid catalyst starting from the alcohol itself in stoichiometric ratio with the thiol; therefore the only byproduct is water. In some cases conversion and selectivity are so high under solvent-free conditions that at the end the product can be separated from the catalyst and purified without any other work up with an E factor = 0.07 for the reaction of **1a** and **2a**. This is quite different from the case of ZnI_2_-catalyzed reaction that requires an excess of thiophenol, use of anhydrous CH_2_Cl_2_ as solvent, quenching of the catalyst with water, double extraction with dichloromethane, washing with brine, drying over sodium sulfate and evaporation of the solvent before chromatographic purification [29]. The reaction does not need to be carried out under inert atmosphere and moreover, the catalyst can be reused several times without significant decrease in selectivity and only a little bit in activity (Figure 6).

The substrate scope is also quite wide as other systems only convert propargylic alcohols [30] or benzhydrol and electron-deficient thiols [31] in agreement with the high electrophilicity of these substrates.

## Conclusion

Amorphous solid acid catalysts are very promising materials in the roadmap to green and sustainable organic synthesis. They allow us to set up very selective processes without producing any waste and avoiding the use of toxic substrates or metals. In particular a 0.6% Al_2_O_3_ on silica, can be conveniently used for the green synthesis of thioethers starting from aromatic alcohols and aromatic or aliphatic thiols. The reaction can be carried out under solvent-free conditions with stoichiometric amounts of the reagents with excellent yield and the catalyst can be reused several times.

## Supporting Information

File 1 Experimental, NMR analysis and copies of spectra.
