# Supplementary material for: Selective synthesis of thioethers in the presence of a transition-metal-free solid Lewis acid
Source: Beilstein J Org Chem. 2016 Dec 6;12:2627–35. doi: 10.3762/bjoc.12.259 (PMC5238612; doi:10.3762/bjoc.12.259)
Supplement: File 1 — Experimental, NMR analysis and copies of spectra. [file Beilstein_J_Org_Chem-12-2627-s001.pdf]

Supporting Information

for

Selective synthesis of thioethers in the presence of a transition metal free solid Lewis acid

Federica Santoro, Matteo Mariani, Federica Zaccheria, Rinaldo Psaro and Nicoletta Ravasio*

Address: CNR ISTM, via C. Golgi 19, 20133 Milano, Italy

Email: Nicoletta Ravasio – n.ravasio@istm.cnr.it

* Corresponding author

Experimental, NMR analysis and copies of spectra

Table of Contents

Materials	S3
Experimental procedure for S-alkylation reactions and some results	S3
Attribution of ^{1}H NMR and ^{13}C NMR signals for some selected compounds	S7
Copies of ^{1}H NMR and ^{13}C NMR spectra	S12
Copies of mass spectra	S20
Optical rotatory power determination	S24

Materials

All the substrates were supplied by Sigma-Aldrich and used without any further manipulation. For the reactions carried out in solvent, toluene was supplied by Sigma-Aldrich and was used as received. The features and the suppliers of the supports and the copper catalysts are reported in Table S1.

Thermogravimetric analysis (TGA) to determine the surface hydroxy groups number was performed on Perkin Elmer 7 HT thermobalance. The analyses were performed by heating the sample from 50 °C to 1,000 °C with a temperature ramp of 5 °C/min. The water loss was evaluated in the range of 250–900 °C.

Table S1: Textural properties of solid acid catalysts.

Solid acid catalyst	Acronym	Supplier	Co-oxide loading (wt %)	BET (m^2/g)	PV (mL/g)
SiO_2-Al_2O_3 0.6	SiAl 0.6	Grace Davison	0.6	488	1.43
SiO_2-Al_2O_3 135	SiAl 13	Sigma-Aldrich	13	485	0.79
SiO_2-ZrO_2 4.7	SiZr 4.7	Grace Davison	4.7	421	2.38
SiO_2-TiO_2 2.3	SiTi 2.3	Grace Davison	2.3	297	1.26

Experimental procedure for S-alkylation of alcohols in solvent

In a typical catalytic test, a mixture of alcohol (0.8 mmol) and thiol (0.8 mmol) were dissolved in toluene (8 mL), and the solution transferred under N_2 or atmospheric air into a glass reaction vessel in which the solid acid catalyst, if necessary, had been properly pretreated (100 mg). Reactions were carried out under atmospheric pressure of N_2 or atmospheric air and constant magnetic stirring (1000 rpm) by using a Teflon-coated magnetic stirring bar at the appropriate temperature (oil bath temperature). After reaction, the mixture has been decanted and the product and the catalyst filtered off by

a PTFE syringe filter. Solvent has been distilled under reduced pressure, and reaction mixtures were analyzed by GC–MS (5% phenylmethyl polysiloxane capillary column length 30 m, injection T = 60 °C) and by ^{1}H NMR and ^{13}C NMR spectroscopy. The NMR spectra reported below have been registered without any further purification.

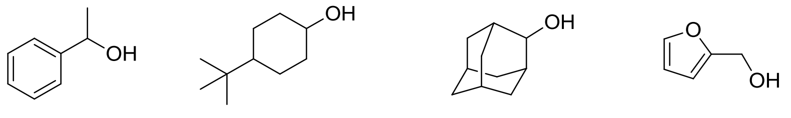

1j **1k** **1l** **1m**

Figure S1: Structures of alcohols not discussed in the manuscript **1j-m**.

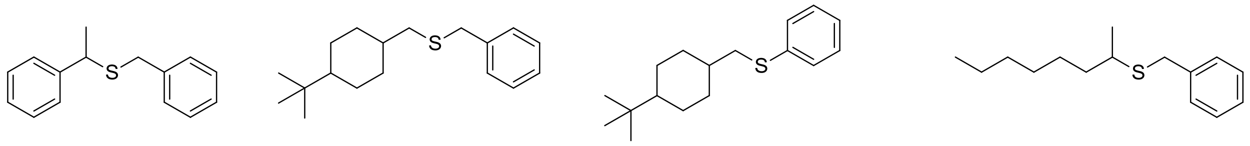

3q **3r** **3s** **3t**

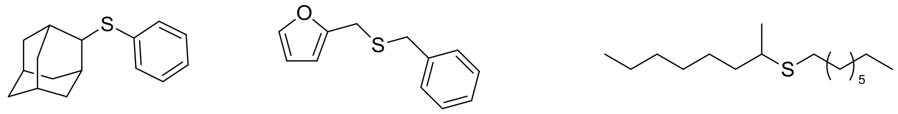

3u **3v** **3w**

Figure S2: Structures of thioethers not discussed in the manuscript **3q-w**.

Table S2: Some results in the synthesis of thioethers from different alcohols and thiols promoted by SiAl 0.6 in solvent[a].

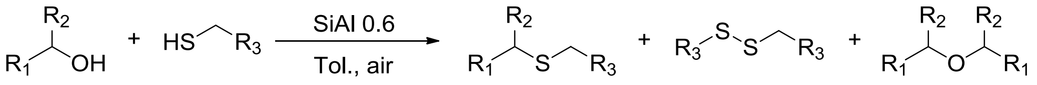

1a-m **2a-d** **3a-v** **4a-d** **5a-m**

Entry	**1a–m**	**2a–d**	T^{b} (°C)	t (h)	Conv. (%)	**3a–v** (%)	**4a–d** (%)	**5a–m** (%)	Sel. Dehy.[c] (%)
1	**1a**	**2a**	60	1	>99.0	**3a** >99.0	**4a** –	**5a** –	–
2[d]	**1a**	**2a**				**3a**	**4a**	**5a**	

			60	1	>99.0	90.8	0.5	7.7	1.0
3	**1a**	**2a**				**3a**	**4a**	**5a**	
			80	0.5	>99.0	>99.0	–	–	<1.0
4[e]	**1a**	**2b**				**3b**	**4b**	**5a**	
			60	5	16.1	–	21.5	15.6	61.1
5	**1a**	**2b**				**3b**	**4b**	**5a**	
			60	0.5	98.5	82.0	1.0	13.3	3.7
				1	>99	94.7	<1.0	1.7	3.2
6	**1a**	**2c**				**3c**	**4c**	**5a**	
			60	0.5	>99	>99	–	–	–
7	**1a**	**2d**				**3d**	**4d**	**5a**	
			60	0.5	>99	>99	–	–	–
8	**1j**	**2a**				**3q**	**4a**	**5j**	
			60	0.5	5.1	0	44.4	–	55.6
				5	8.3	0	13.2	40.1	46.7
9	**1j**	**2a**				**3q**	**4a**	**5j**	
			80	0.5	12.2	11.0	9.8	48.4	30.8
				5	47.8	20.9	3.2	68.9	7.0
10	**1c**	**2a**				**3e**	**4a**	**5c**	
			60	1	9.8	28.3	6.4	60.4	–
				4	15.7	34.1	10.3	42.9	–
11	**1c**	**2a**				**3e**	**4a**	**5c**	
			80	0.5	59.8	60.0	2.8	33.9	–
				1	78.4	85.6	–	12.4	–
				2	94.3	96.8	–	1.4	–
12	**1k**	**2a**				**3r**	**4a**	**5k**	
			60	6	–	–	–	–	–

13	**1k**	**2a**	80	7	–	**3r** –	**4a** –	**5k** –	–
14	**1k**	**2b**	110	8	–	**3s** –	**4b** –	**5k** –	–
15	**1f**	**2a**	110	24	–	**3t** –	**4a** –	**5f** –	–
16	**1l**	**2b**	110	12	10.6	**3u** –	**4b** 10.6	**5l** –	–
17	**1m**	**2a**	60	4	–	**3v** –	**4a** –	**5m** –	–
18	**1m**	**2a**	110	0.5	–	**3v** –	**4a** –	**5m** –	–

[a]Reaction conditions: Cat.=100mg, Cat./ROH=1:1 (w/w), ROH/RSH=1:1(mol/mol), toluene (8 mL), air, stirring (1000 rpm); reaction mixtures were analysed by GC–MS (5% phenylmethyl polysiloxane capillary column length 30 m, injection T = 60 °C), and by ^{1}H NMR and ^{13}C NMR spectroscopy; conversion was calculated with respect to the thiol. [b]Oil bath temperature. [c]Corresponding substituted styrene derived from alcohol dehydration. [d]Reaction with cat./ROH = 1:2 (w/w). [e]Reaction without catalyst.

Experimental procedure for S-alkylation of alcohols without solvent

In a typical reaction the alcohol (0.8 mmol) and the thiol (0.8 mmol) were introduced into a glass tube with a screw cap contained SiAl 0.6 (10 mg). Reactions were carried out under atmospheric air and constant magnetic stirring (1000 rpm) using a Teflon-coated magnetic stir bar at the chosen temperature (oil bath temperature). After reaction, the mixture has been decanted and the product and the catalyst filtered off by a PTFE syringe filter. Reaction mixtures were analysed by GC–MS (5% phenylmethyl polysiloxane capillary column length 30 m, injection T = 60 °C) and by ^{1}H NMR and ^{13}C NMR spectroscopy. The NMR spectra reported below have been registered without any

further purification. The product obtained from the addition of benzylmercaptan **2a** to cinnamyl alcohol **1i** has been identified by comparison with NMR spectra reported in the literature (Angew. Chem. Int. Ed. 2005, 44, 794–797).

Attribution of ^{1}H NMR and ^{13}C NMR signals for some selected compounds

^{1}H NMR and ^{13}C NMR data were recorded on Bruker 300 MHz and 400 MHz spectrometers at ambient temperature. ^{1}H NMR data are presented as follows: chemical shift in ppm relative to internal TMS (0.00 ppm) (multiplicity, coupling constant, integration). The following abbreviations are used in reporting NMR data: s, singlet; d, doublet; t, triplet; q, quartet; dd, doublet of doublets; m, multiplet.

^{13}C NMR data are presented as follows: chemical shift in ppm relative to $CDCl_3$ (77.0 ppm)

Compound 3a: benzyl(1-(4-methoxyphenyl)ethyl)sulfane

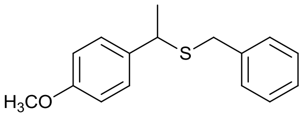

Pale yellow liquid.

^{1}H NMR (300 MHz, $CDCl_3$, δ ppm): 7.37 – 7.20 (m, 7H), 6.97-6.81 (m, 2H), 3.87-3.76 (m, 4H), 3.65-3.42 (m, 2H), 1.54 (d, J = 7.1 Hz, 3H).

$^{13}C\{H\}$ APT NMR (75 MHz, $CDCl_3$, δ ppm): 159.0, 138.9, 136.2, 129.3, 128.9, 128.8, 127.2, 114.2, 55.7, 43.3, 36.0, 231.

[M]+: m/z calcd: 258.11 ; found: 258.1, 135.1, 120.0, 105.0, 91.0, 77.0, 65.0, 59.0.

Compound 3b: (1-(4-methoxyphenyl)ethyl)(phenyl)sulfane

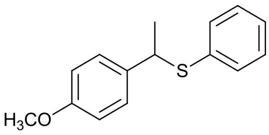

Colourless liquid

^{1}H NMR (300 MHz, $CDCl_3$, δ ppm): 7.33 – 7.20 (m, 7H), 6.90 – 6.73 (m, 2H), 4.35 (q, J = 7.0 Hz, 1H), 3.81 (s, 3H), 1.63 (d, J = 7.0 Hz, 3H).

^{13}C{H} APT NMR (75 MHz, $CDCl_3$, δ ppm): 159.0, 135.6, 132.9, 129.5, 129.1, 128.7, 127.4, 114.2, 55.6, 47.8, 22.8.

[M]+: m/z calcd: 244.09 ; found: 244.0, 135.0, 120.0, 105.0, 91.0.

Ref. Synthetic Communications1, 41: 100–112, 2011.

Compound 3c: (1-(4-methoxyphenyl)ethyl)(naphthalen-2-yl)sulfane

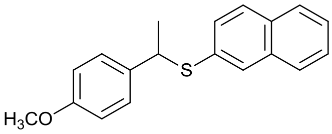

White solid

^{1}H NMR (300 MHz, $CDCl_3$, δ ppm): δ 7.85-7.70 (m, 4H), 7.50 – 7.44 (m, 2H), 7.41 (dd, J = 8.6, 1.6 Hz, 1H), 7.29 (d, J = 8.6 Hz, 2H), 6.84 (d, J = 8.7 Hz, 2H), 4.48 (q, J = 7.0 Hz, 1H), 3.80 (s, 3H), 1.68 (d, J = 7.0, 3H).

^{13}C{H} APT NMR (75 MHz, $CDCl_3$, δ ppm): 159.1, 135.6, 134.0, 133.3, 132.7, 131.4, 130.2, 128.8, 128.5, 128.0, 127.8, 126.7, 126.4, 114.2, 55.7, 47.7, 22.9.

[M]+: m/z calcd: 294.11 ; found: 294.1, 160.0, 135.0, 128.1, 115.1, 102.1, 91.1, 79.1, 65.0.

Compound 3d: cyclohexyl(1-(4-methoxyphenyl)ethyl)sulfane

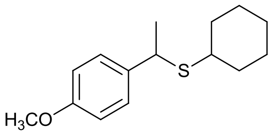

Pale yellow liquid

^{1}H NMR (300 MHz, $CDCl_3$, δ ppm): 7.29 (d, J = 8.7 Hz, 2H), 6.86 (d, J = 8.7 Hz, 2H), 4.04 (q, J = 7.0 Hz, 1H), 3.82 (s, 3H), 2.57 – 2.29 (m, 1H), 1.98 (d, J = 12.7 Hz, 1H), 1.74 (s, 3H), 1.59 (dd, J = 29.4, 23.8 Hz, 5H), 1.29 – 0.86 (m, 4H).

^{13}C{H} APT NMR (75 MHz, $CDCl_3$, δ ppm): 158.8, 137.2, 128.5, 114.2, 55.6, 43.1, 42.2, 34.3, 33.7, 26.4, 26.3, 23.6.

[M]+: m/z calcd: 250.14 ; found: 250.1, 135.0, 120.0, 105.0, 91.0.

Compound 3e: benzyl(4-methoxybenzyl)sulfane

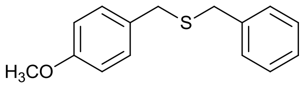

Colourless liquid

^{1}H NMR (300 MHz, $CDCl_3$, δ ppm):7.36 – 7.27 (m, 5H), 7.25 - 7.20 (m, 2H), 6.91 – 6.83 (m, 2H), 3.83 (s, 3H), 3.61 (d, *J* = 9.9 Hz, 4H).

^{13}C{H} APT NMR (75 MHz, $CDCl_3$, δ ppm):159.0, 138.7, 130.5, 129.4, 128.9, 127.3, 114.3, 55.7, 35.9, 35.4.

[M]+: m/z calcd: 244.09 ; found: 244.1, 121.0, 109.0, 91.0, 77.0, 65.0, 51.0.

Ref. *Chem. Eur. J.* **2009**, 15, 793 – 797

Compound 3f: (1-(4-methoxyphenyl)ethyl)(octyl)sulfane

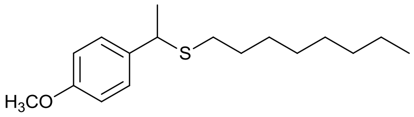

Colourless liquid

^{1}H NMR (300 MHz, $CDCl_{3,}$ δ ppm): 7.31 – 7.24 (m, 2H), 6.91 – 6.84 (m, 2H), 3.94 (q, J = 7.0 Hz, 1H), 3.82 (s, 3H), 2.41 – 2.21 (m, 2H), 1.61 – 1.43 (m, 5H), 1.41 – 1.23 (m, 10H), 0.90 (t, J = 6.8 Hz, 3H).

^{13}C{H} ZGDC NMR (75 MHz, $CDCl_{3,}$ δ ppm): 158.9, 136.7, 128.6, 114.2, 55.6, 43.8, 32.2, 31.7, 29.8, 29.6, 29.5, 29.4, 23.2, 23.0, 14.5.

[M]+: m/z calcd: 280.19 ; found: 280.1, 135.1, 120.0, 105.0, 91.0, 77.0, 65.0, 55.0.

Compound 3h: benzyl(1-(*p*-tolyl)ethyl)sulfane

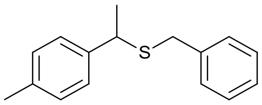

Pale yellow liquid

^{1}H NMR (300 MHz, $CDCl_{3,}$ δ ppm): 7.39 – 7.14 (m, 9H), 3.83 (q, *J* = 7.0 Hz, 1H), 3.53 (m, 2H), 2.39 (s, 3H), 1.56 (d, *J* = 7.1 Hz, 3H).

^{13}C{H} APT NMR (75 MHz, $CDCl_3$, δ ppm):141.2, 138.9, 137.1, 129.6, 129.3, 128.8, 127.8, 127.2, 43.7, 36.1, 23.0, 21.5.

$[M]^{+}$: m/z calcd: 242.11; found: 242.1, 119.1, 103.0, 91.0, 77.0, 65.0, 59.0, 51.0.

Compound 3j: benzyl(2-(*p*-tolyl)propan-2-yl)sulfane

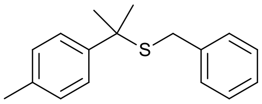

Colourless-pale yellow liquid

^{1}H NMR (400 MHz, $CDCl_3$, δ ppm): 7.51 (d, J = 8.0 Hz, 2H), 7.29 – 7.17 (m, 7H), 3.45 (s, 2H), 2.40 (s, 3H), 1.75 (s, 6H).

$^{13}C\{H\}$ APT NMR (100 MHz, $CDCl_3$, δ ppm):143.3, 138.3, 136.1, 128.9, 128.9, 128.3, 126.7, 126.6, 111.6, 48.4, 34.6, 30.3, 20.96.

$[M]^+$: m/z calcd: 256.13; found: 256.1, 133.1, 124.0, 117.0, 105.1, 91.0, 77.0, 65.0, 51.0.

Ref. *Chem. Commun.* **2016**, 52, 8291-8293.

Copies of ^{1}H NMR and ^{13}C NMR spectra

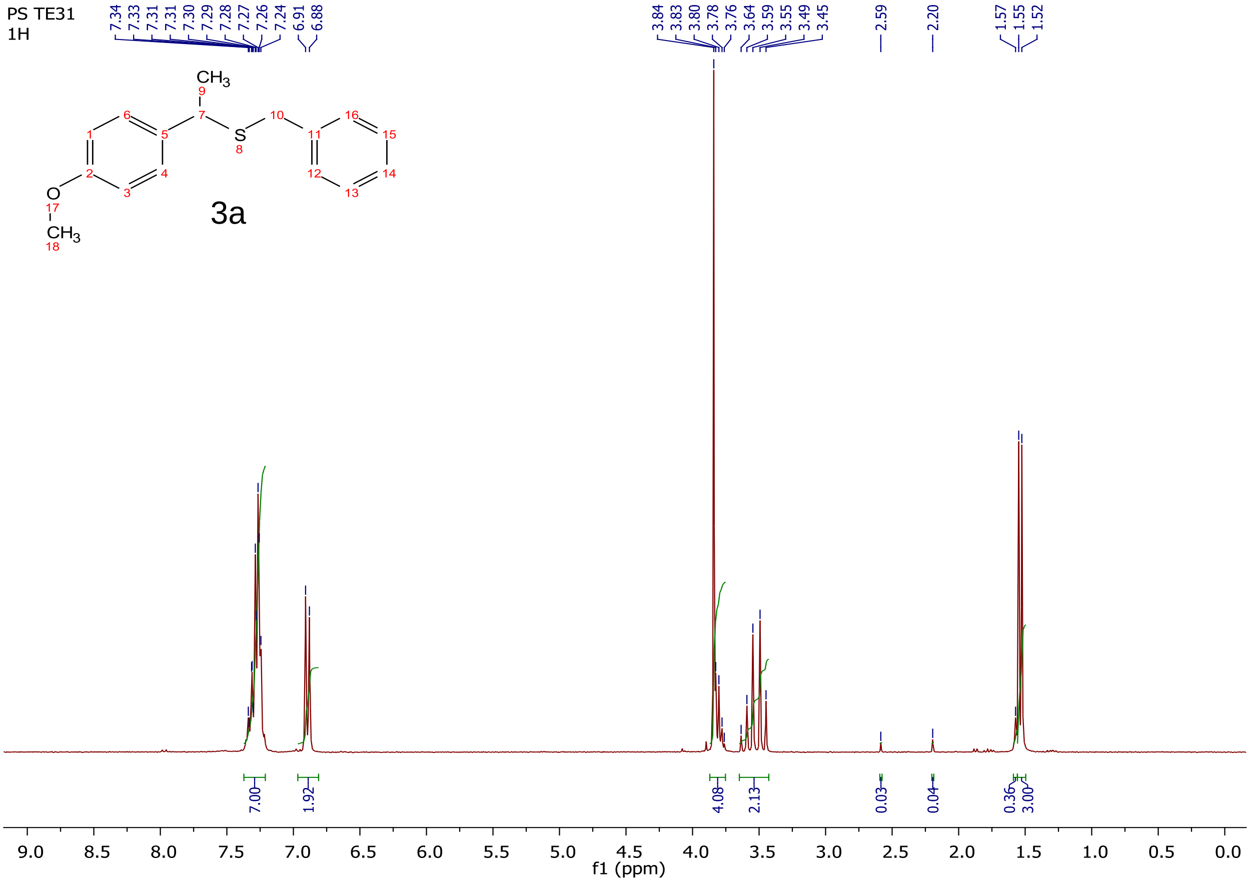

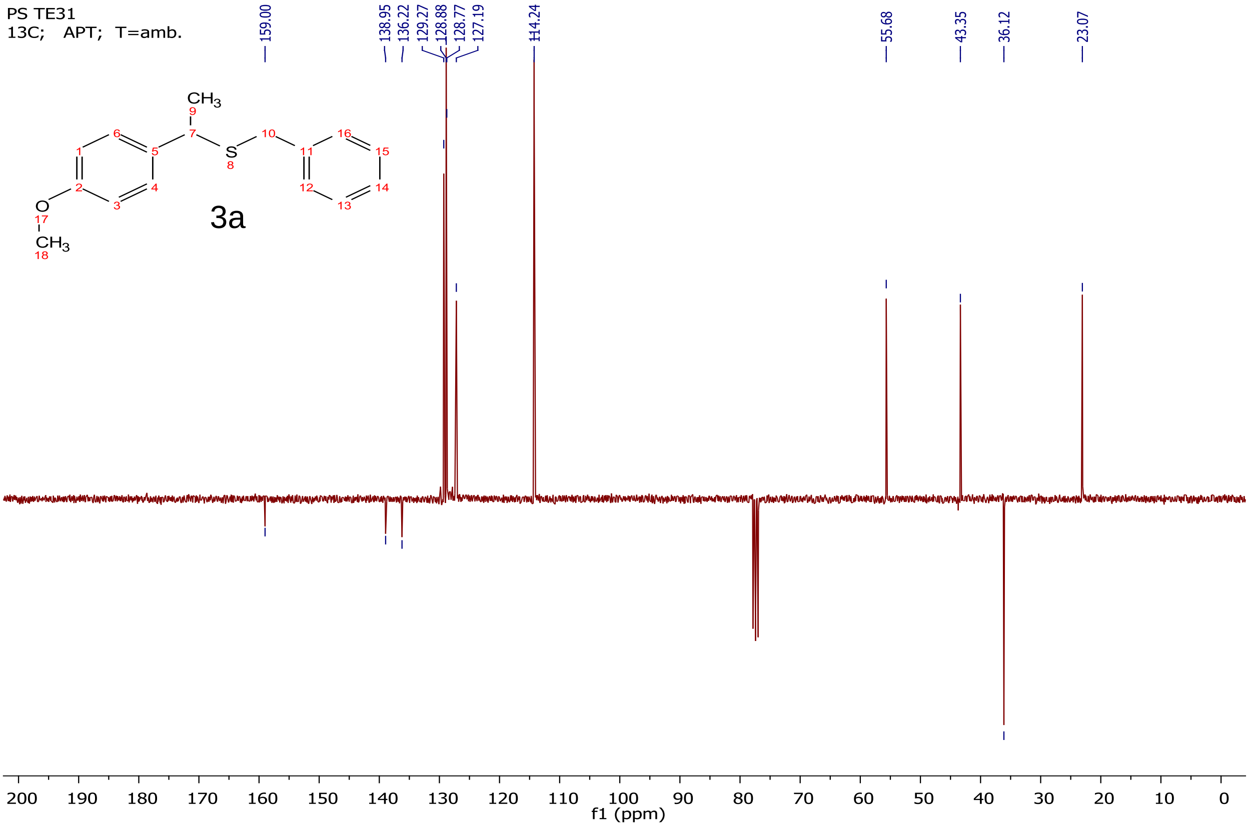

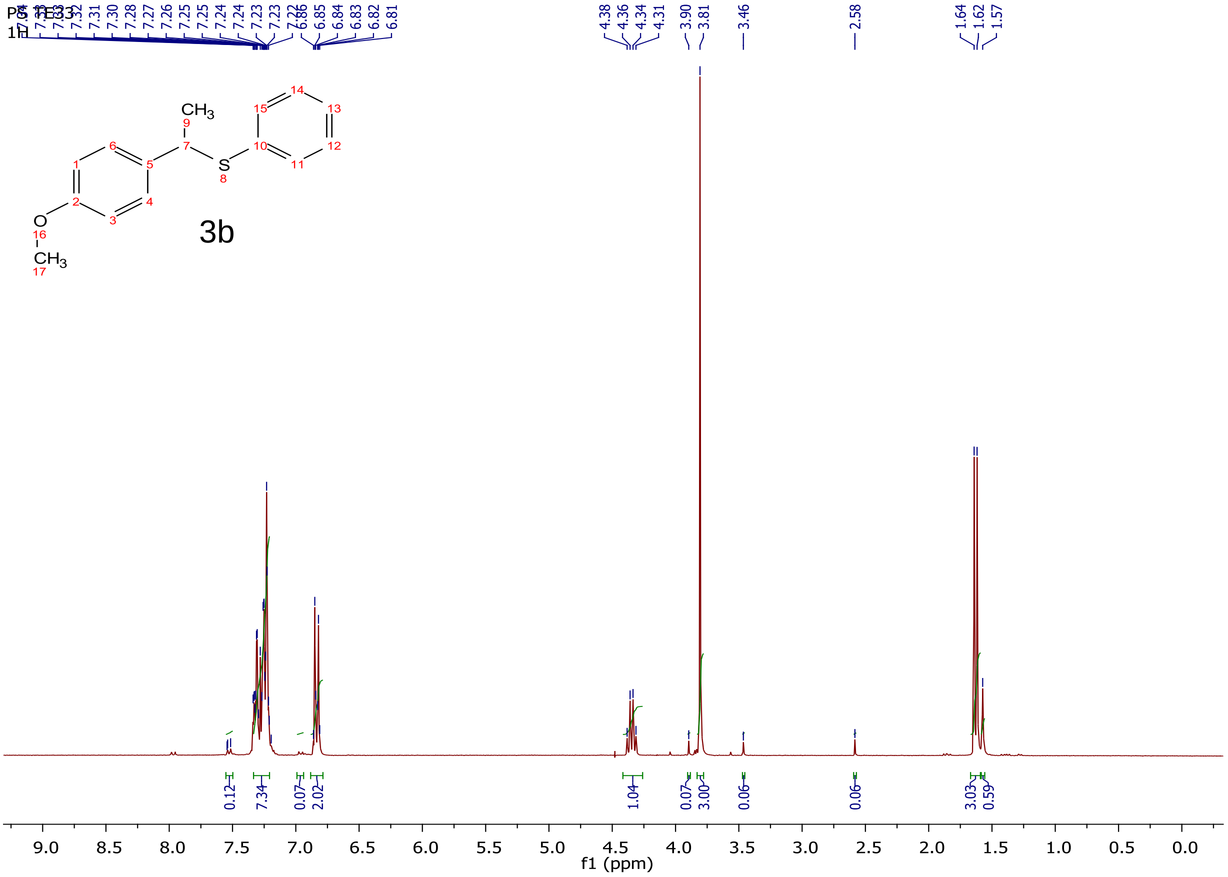
PS TE33
1H
4.38
4.36
4.34
4.31
3.90
3.81
3.46
2.58
1.64
1.62
1.57
3b
0.12
7.34
0.07
2.02
1.04
0.07
3.00
0.06
0.06
3.03
0.59
f1 (ppm)

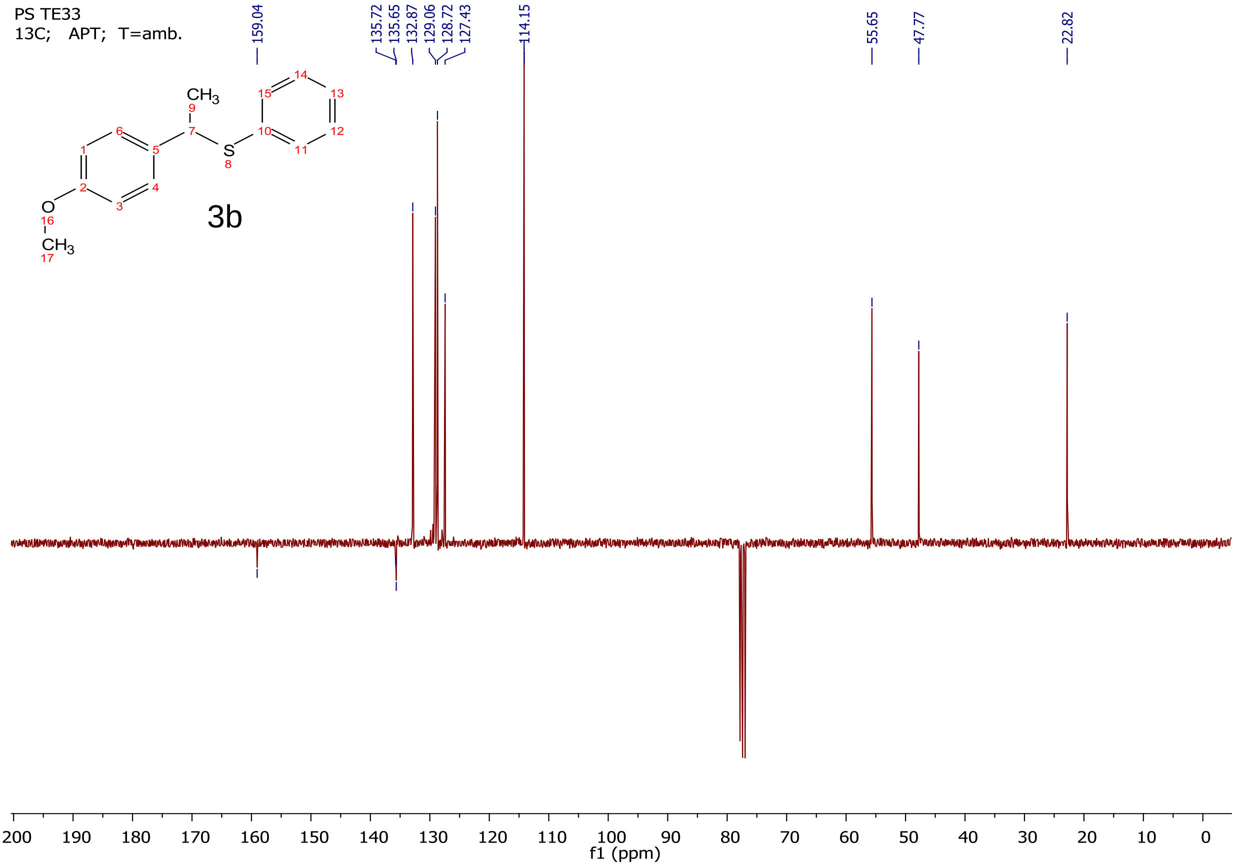
PS TE33
13C; APT; T=amb.
159.04
135.72
135.65
132.87
129.06
128.72
127.43
114.15
55.65
47.77
22.82
3b
f1 (ppm)

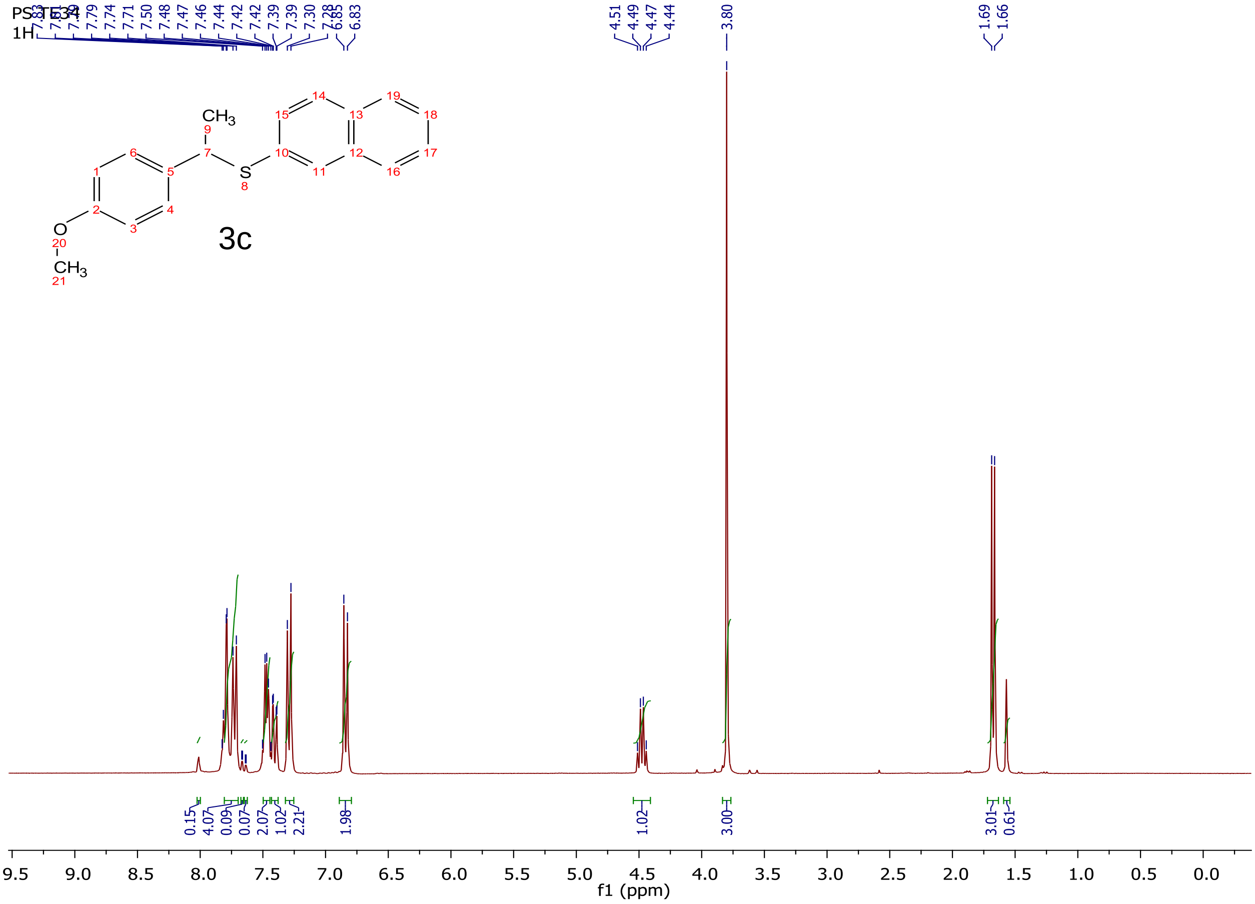
PS TE34
1H
7.79
7.74
7.71
7.50
7.48
7.47
7.46
7.44
7.42
7.42
7.39
7.39
7.30
6.83
4.51
4.49
4.47
4.44
3.80
1.69
1.66
CH3
S
O
CH3
3c
0.15
4.07
0.09
0.07
2.07
1.02
2.21
1.98
1.02
3.00
3.01
0.61
f1 (ppm)

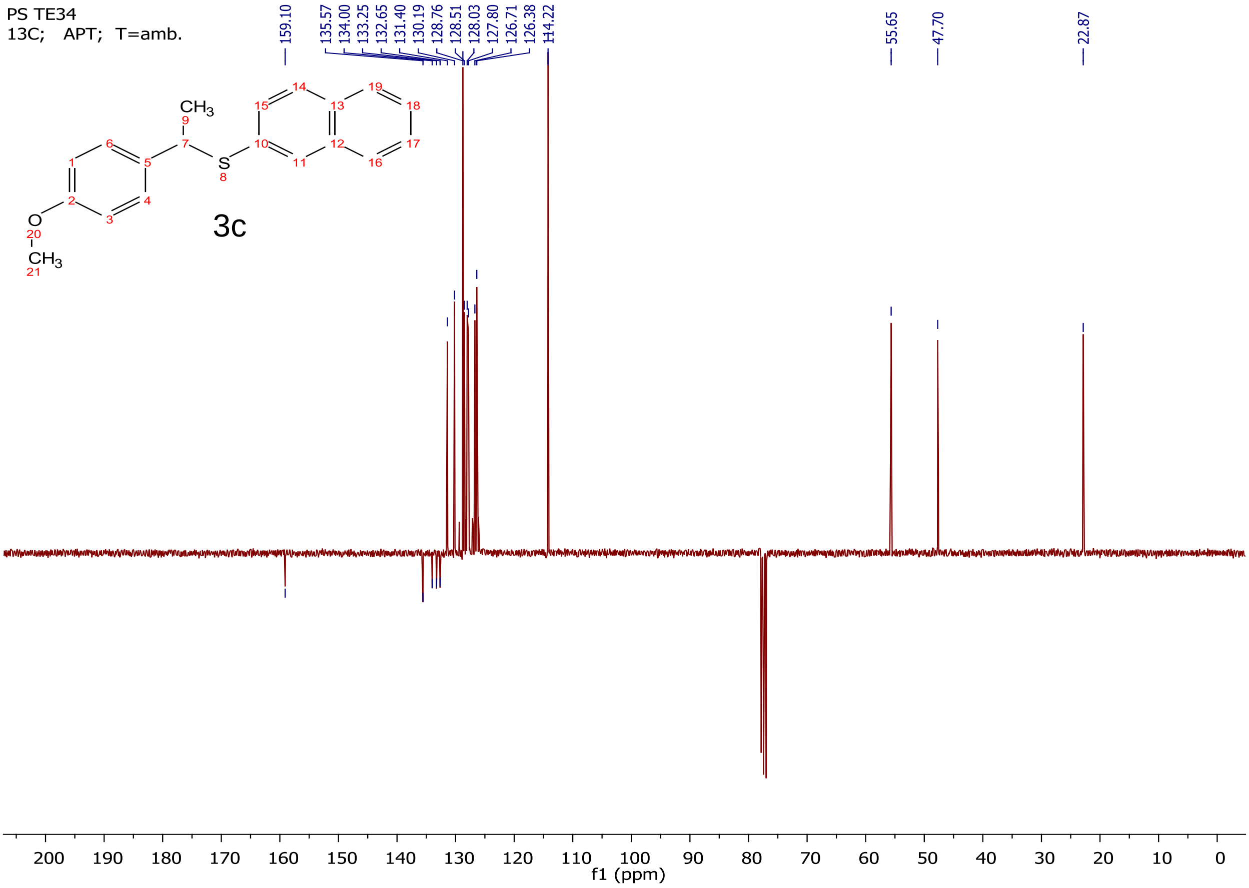
PS TE34
13C; APT; T=amb.
159.10
135.57
134.00
133.25
132.65
131.40
130.19
128.76
128.51
128.03
127.80
126.71
126.38
114.22
55.65
47.70
22.87
CH3
S
O
CH3
3c
f1 (ppm)

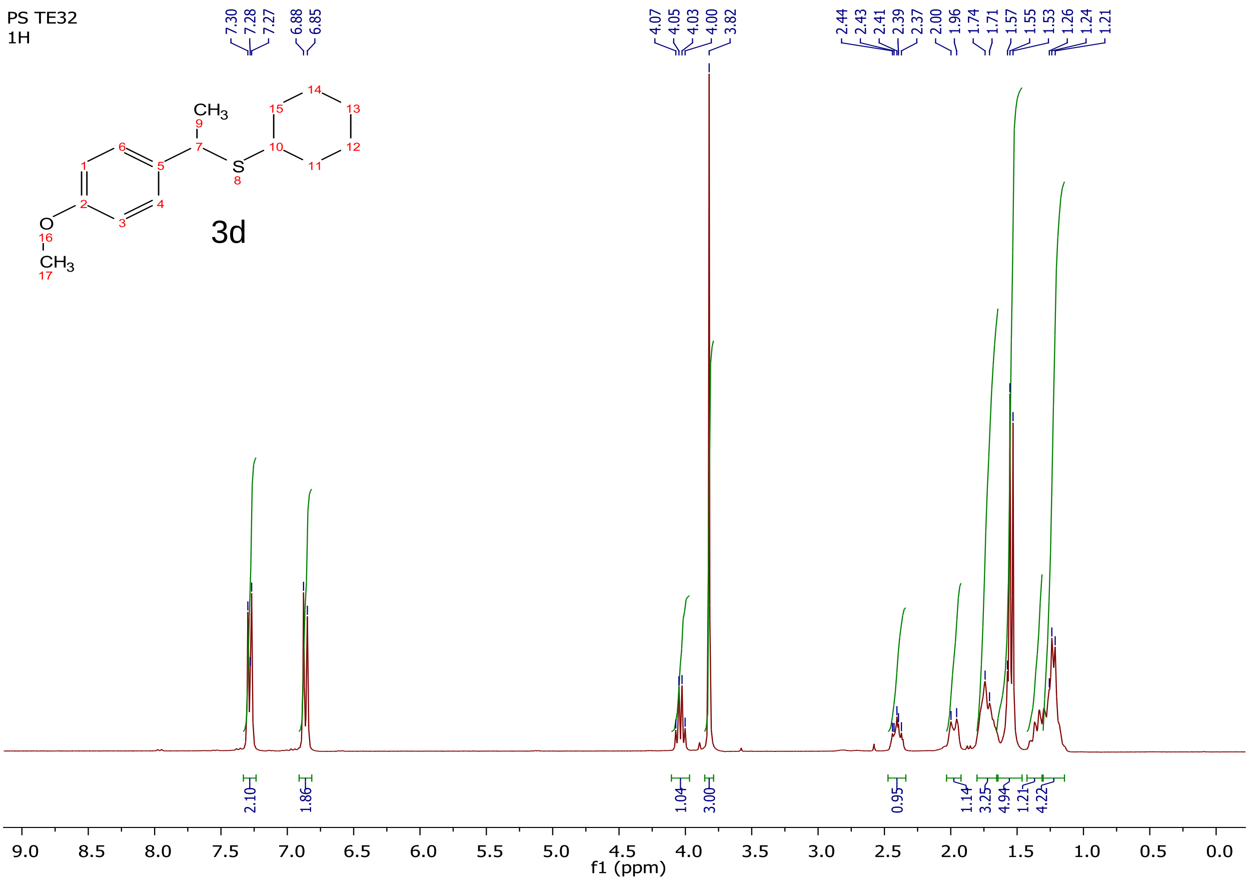
PS TE32
1H
7.30
7.28
7.27
6.88
6.85
4.07
4.05
4.03
4.00
3.82
2.44
2.43
2.41
2.39
2.37
2.00
1.96
1.74
1.71
1.57
1.55
1.53
1.26
1.24
1.21
3d
2.10
1.86
1.04
3.00
0.95
1.14
3.25
4.94
1.21
4.22
f1 (ppm)

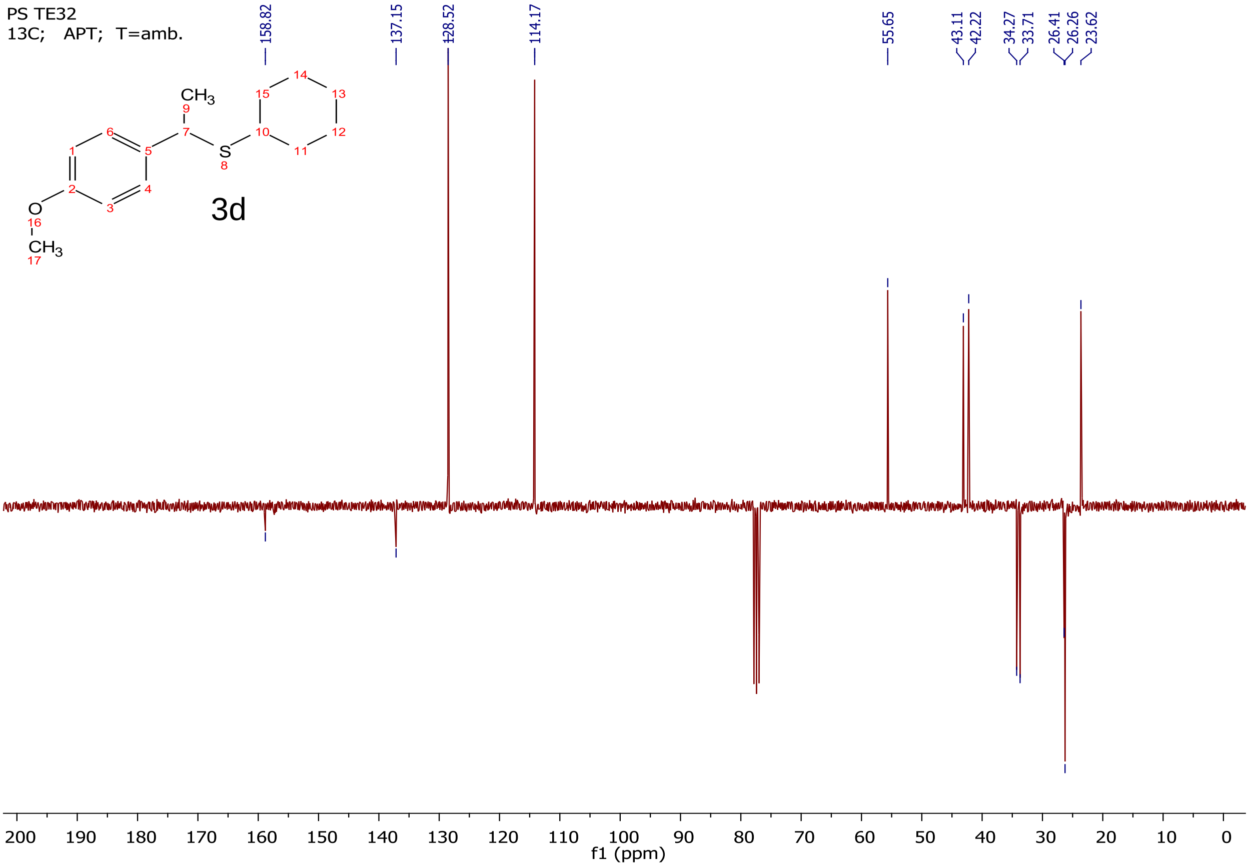
PS TE32
13C; APT; T=amb.
158.82
137.15
128.52
114.17
55.65
43.11
42.22
34.27
33.71
26.41
26.26
23.62
3d
f1 (ppm)

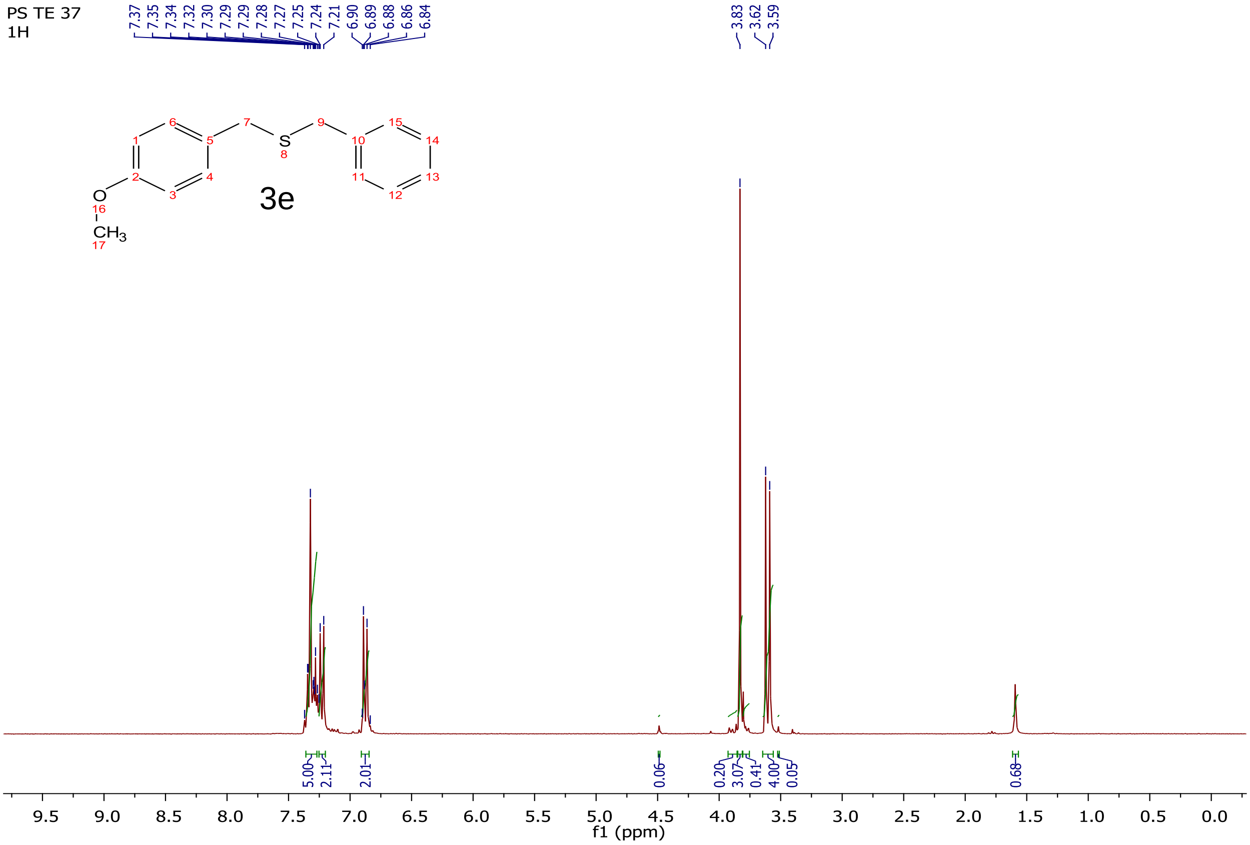
PS TE 37
1H
7.37 7.35 7.34 7.32 7.30 7.29 7.29 7.28 7.27 7.25 7.24 7.21 6.90 6.89 6.88 6.86 6.84
3.83 3.62 3.59
3e
5.00 2.11 2.01 0.06 0.20 3.07 0.41 4.00 0.05 0.68
f1 (ppm)

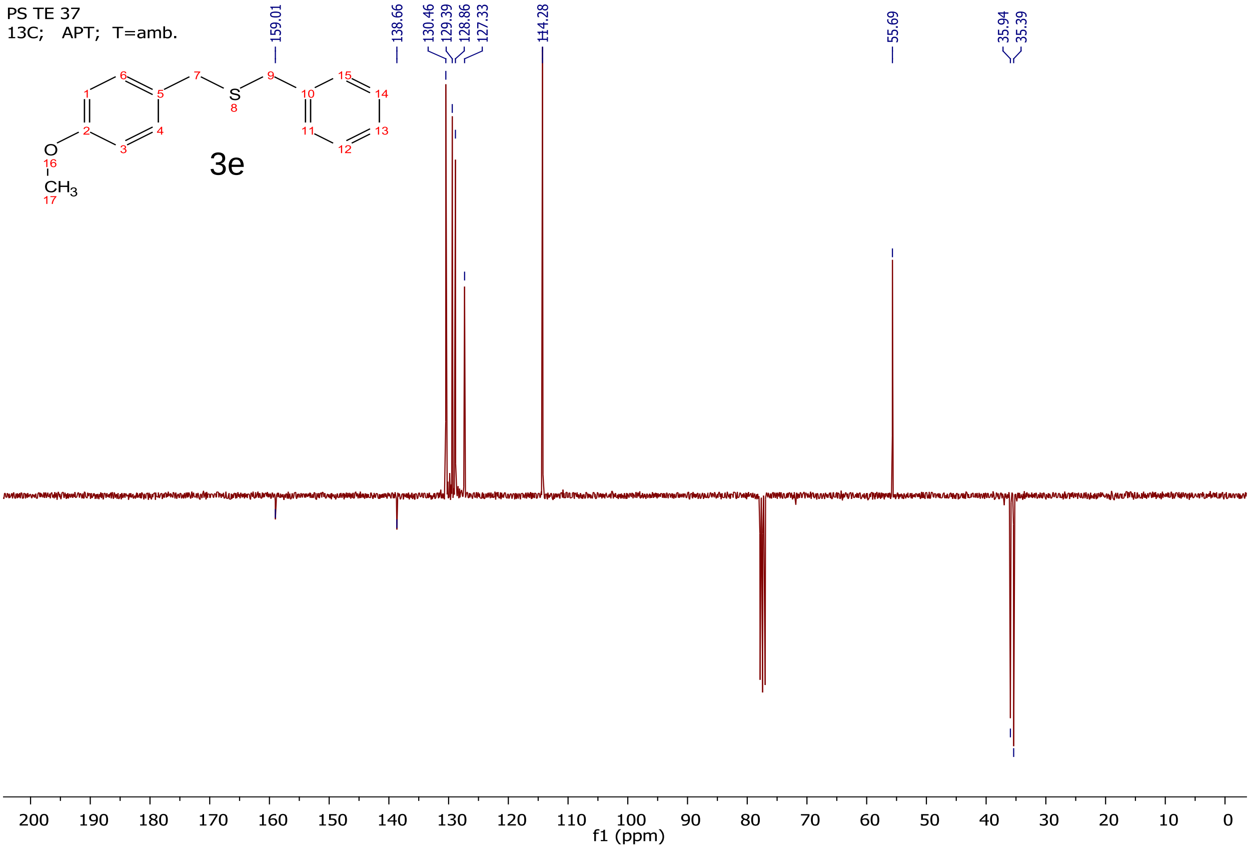
PS TE 37
13C; APT; T=amb.
159.01 138.66 130.46 129.39 128.86 127.33 114.28 55.69 35.94 35.39
3e
f1 (ppm)

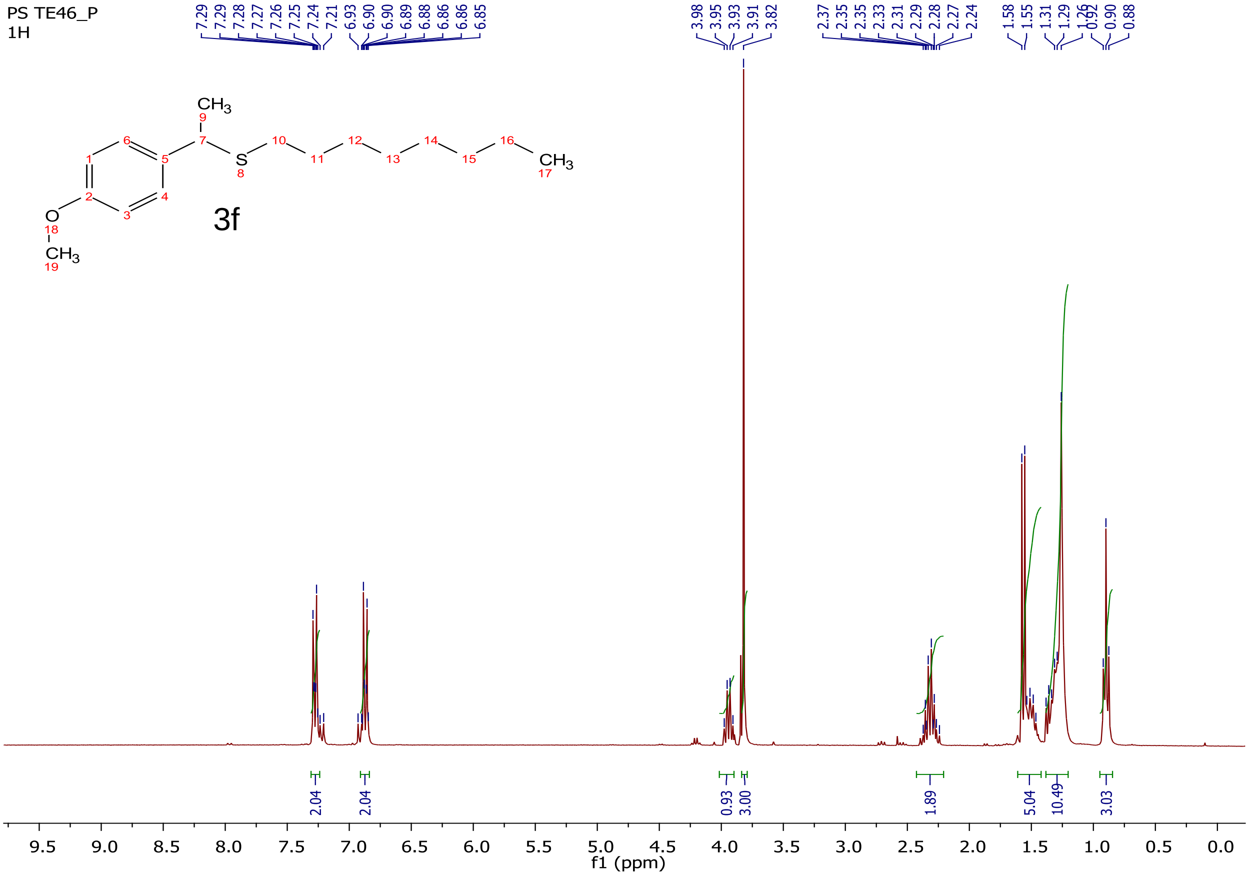
PS TE46_P
1H
7.29
7.29
7.28
7.27
7.26
7.25
7.24
7.21
6.93
6.90
6.90
6.89
6.88
6.86
6.86
6.85
3.98
3.95
3.93
3.91
3.82
2.37
2.35
2.35
2.33
2.31
2.29
2.28
2.27
2.24
1.58
1.55
1.31
1.29
1.26
0.92
0.90
0.88
3f
2.04
2.04
0.93
3.00
1.89
5.04
10.49
3.03
f1 (ppm)

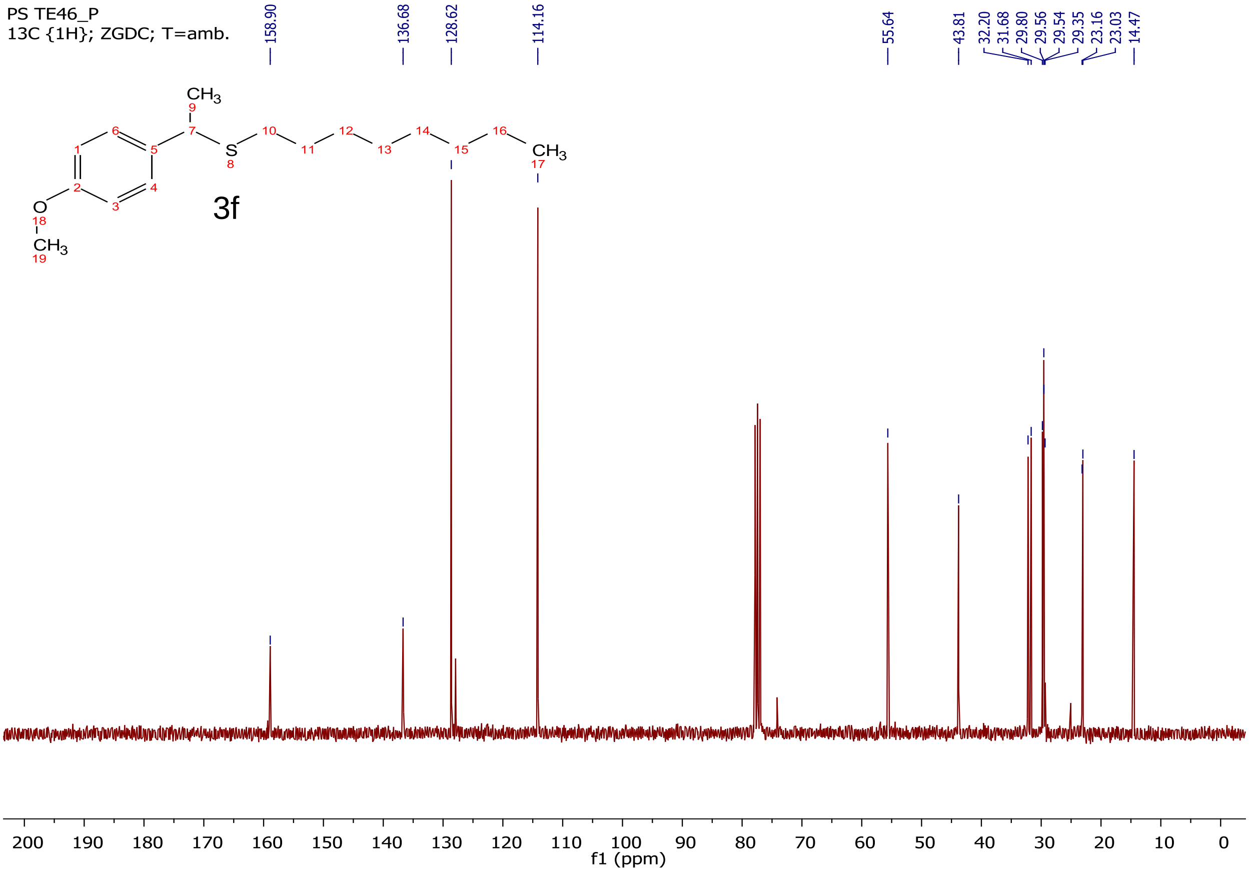
PS TE46_P
13C {1H}; ZGDC; T=amb.
158.90
136.68
128.62
114.16
55.64
43.81
32.20
31.68
29.80
29.56
29.54
29.35
23.16
23.03
14.47
3f
f1 (ppm)

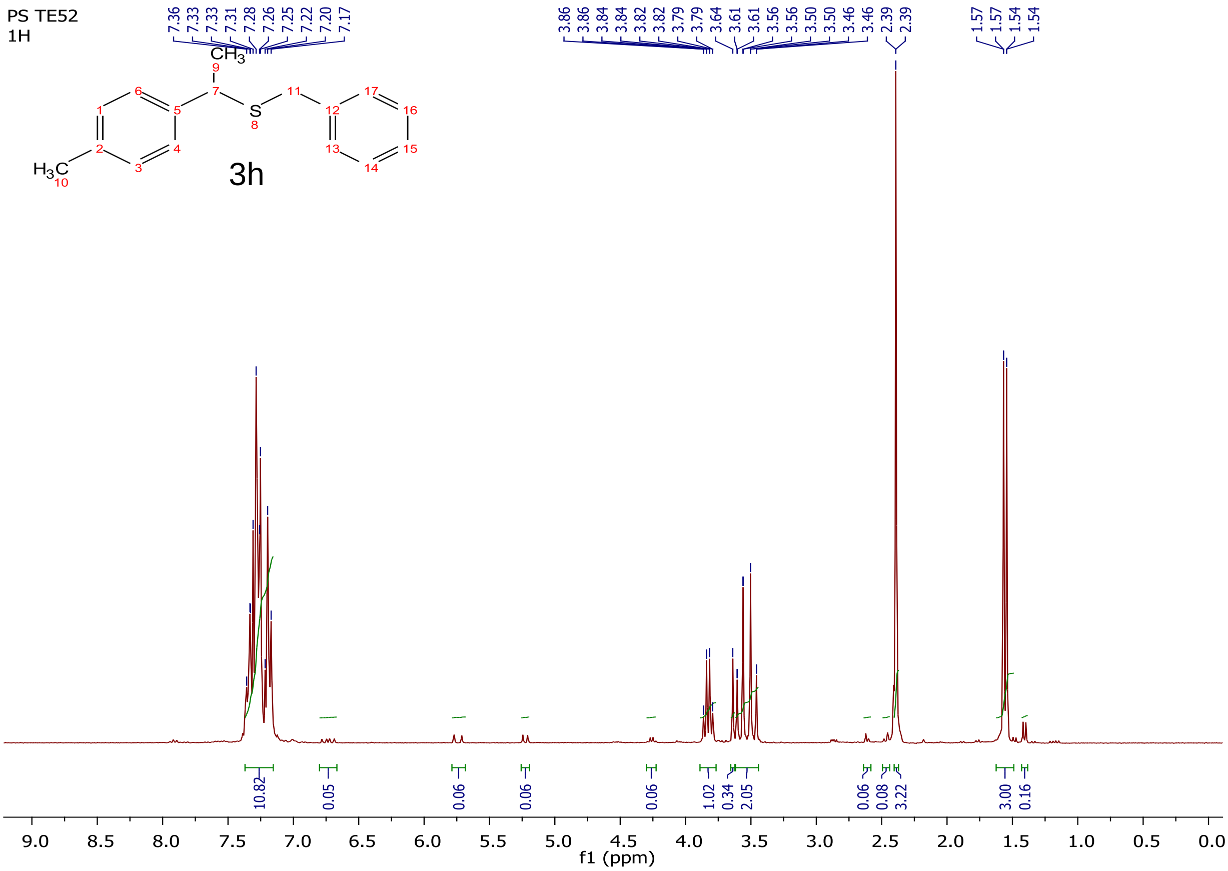
PS TE52
1H
7.36 7.33 7.33 7.31 7.28 7.26 7.25 7.22 7.20 7.17
3.86 3.86 3.84 3.84 3.82 3.82 3.79 3.79 3.64 3.61 3.61 3.56 3.56 3.50 3.50 3.46 3.46 2.39 2.39
1.57 1.57 1.54 1.54
3h
10.82 0.05 0.06 0.06 0.06 1.02 0.34 2.05 0.06 0.08 3.22 3.00 0.16
f1 (ppm)

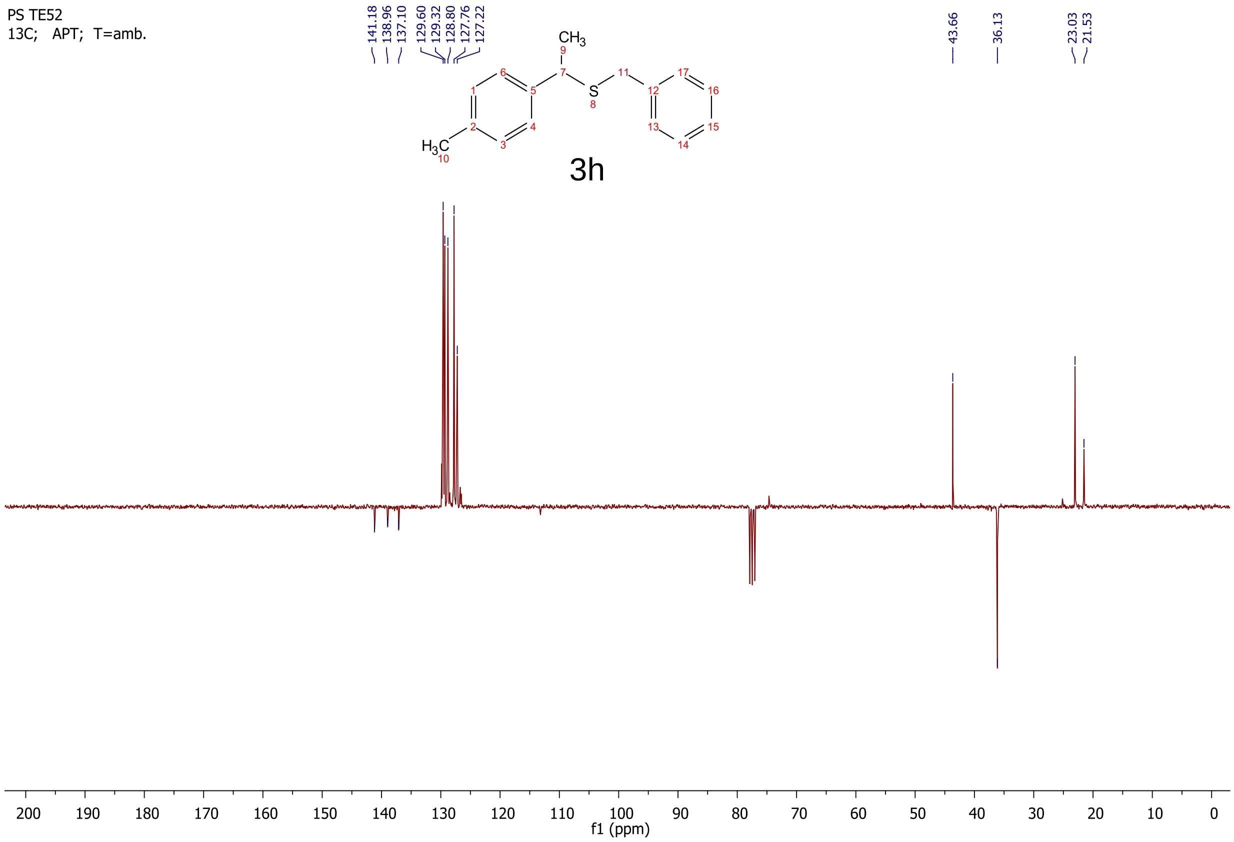
PS TE52
13C; APT; T=amb.
141.18 138.96 137.10 129.60 129.32 128.80 127.76 127.22
43.66
36.13
23.03 21.53
3h
f1 (ppm)

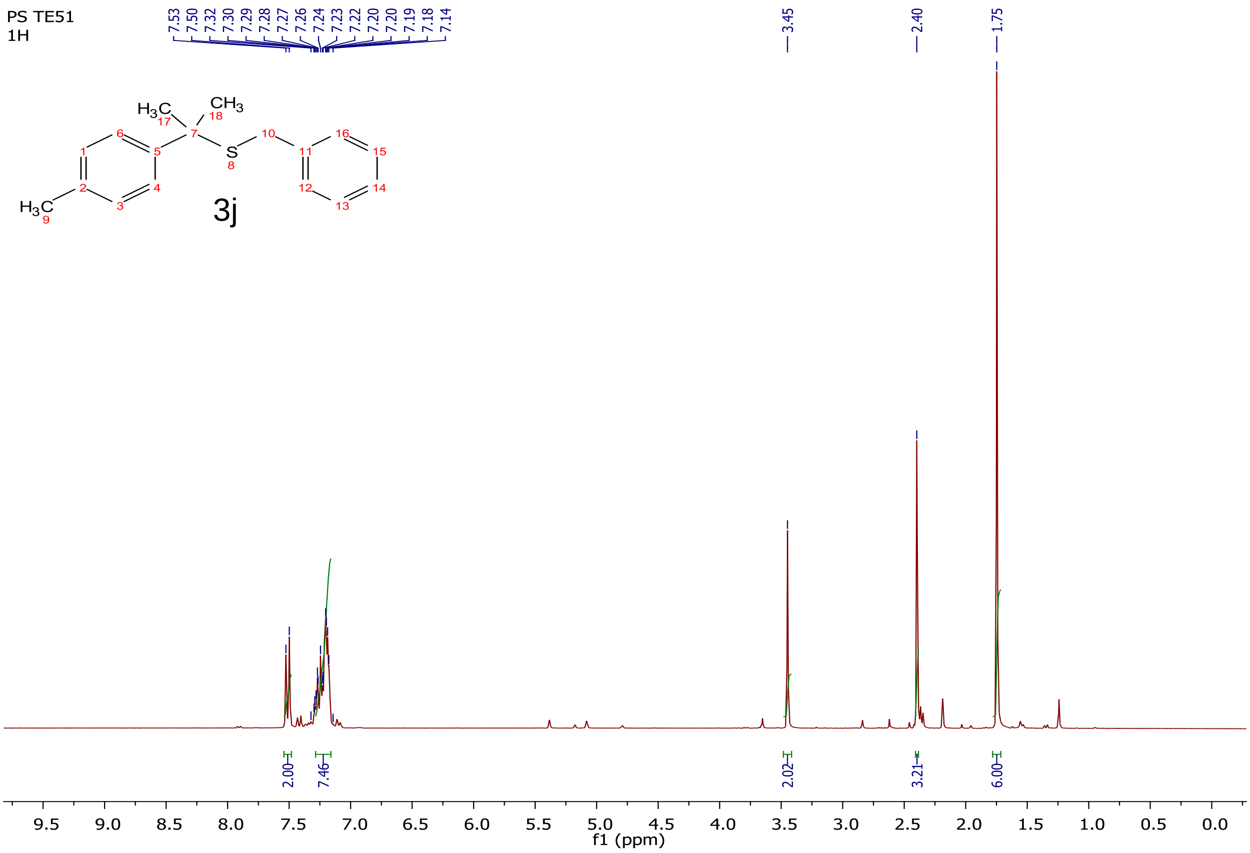
PS TE51
1H
7.53 7.50 7.32 7.30 7.29 7.28 7.27 7.26 7.24 7.23 7.22 7.20 7.19 7.18 7.14
3.45
2.40
1.75
3j
2.00
7.46
2.02
3.21
6.00
f1 (ppm)

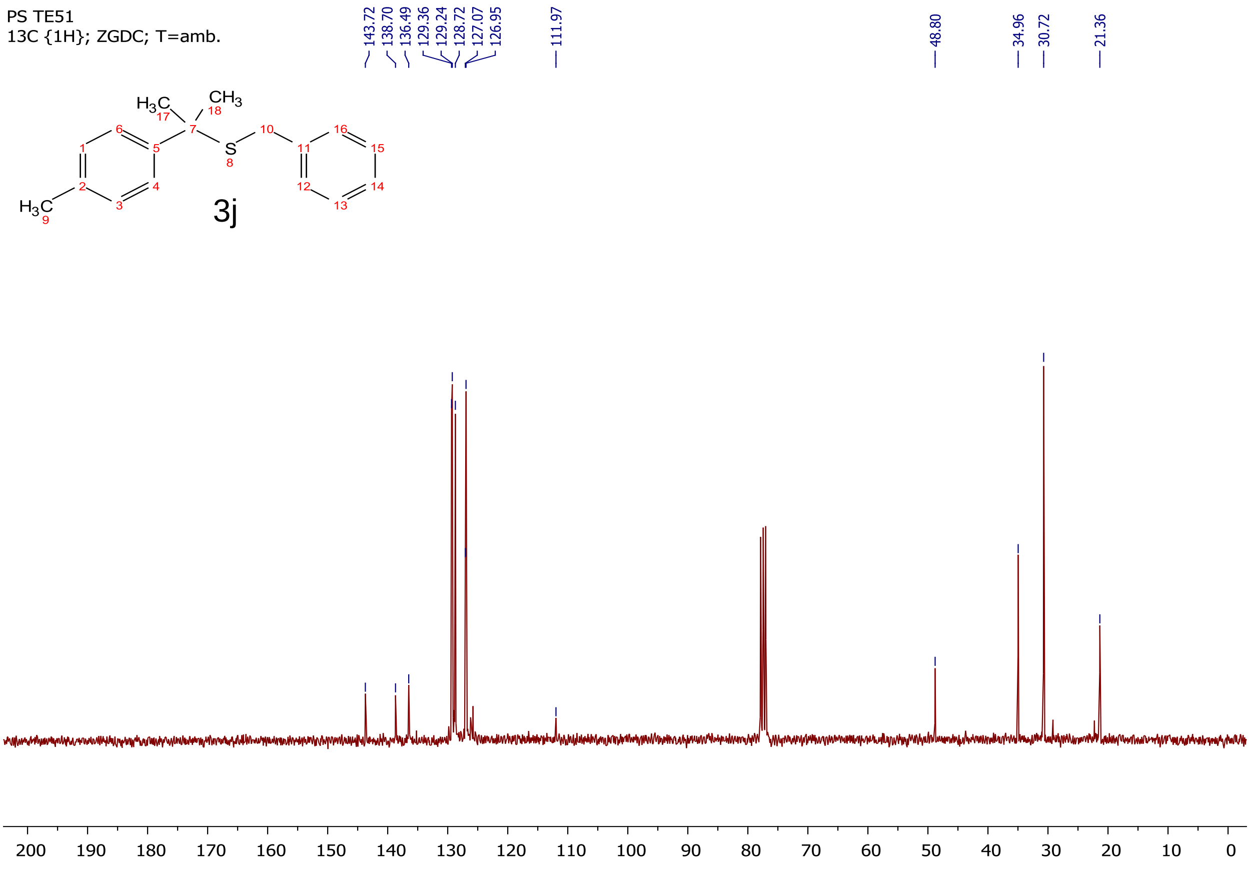
PS TE51
13C {1H}; ZGDC; T=amb.
143.72 138.70 136.49 129.36 129.24 128.72 127.07 126.95
111.97
48.80
34.96
30.72
21.36
3j

Copies of mass spectra

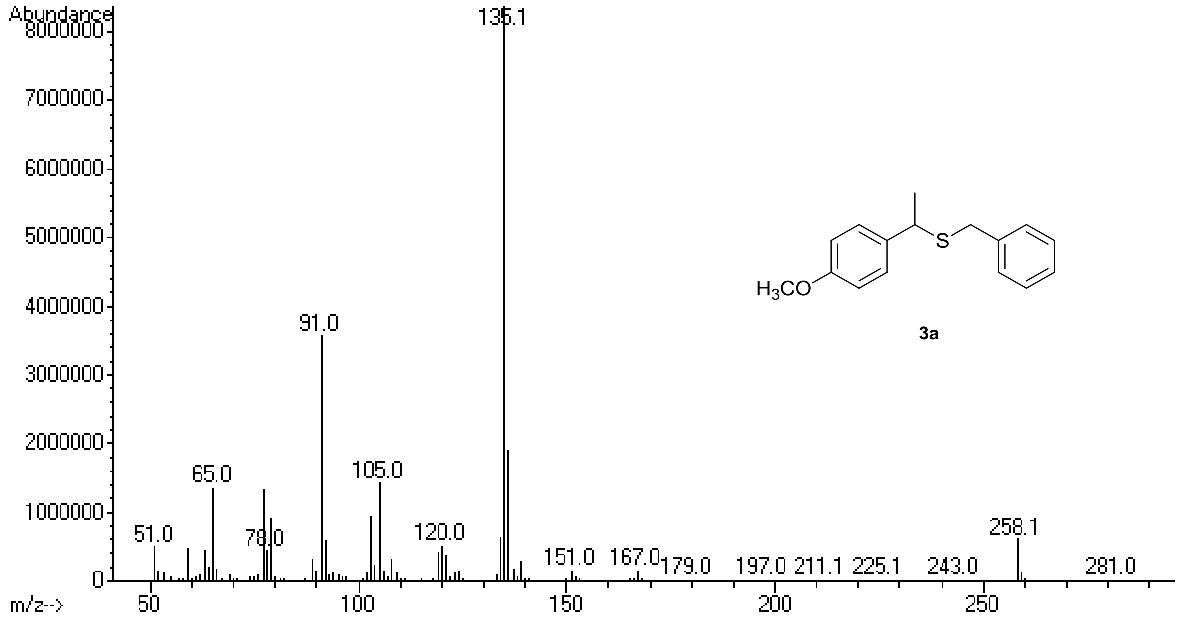

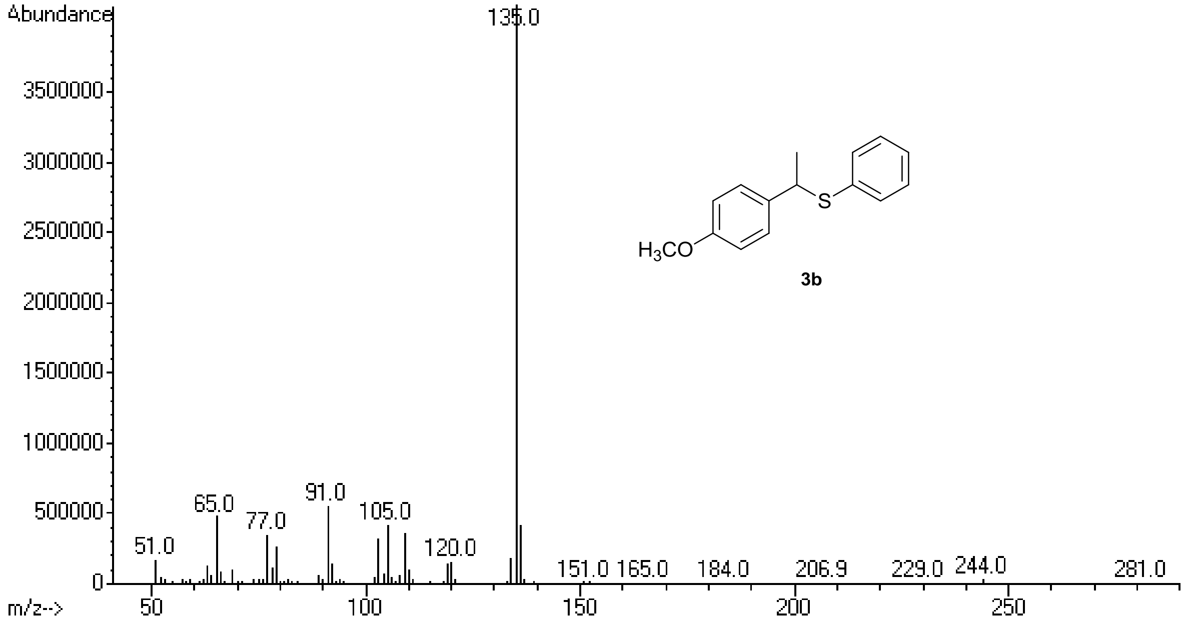

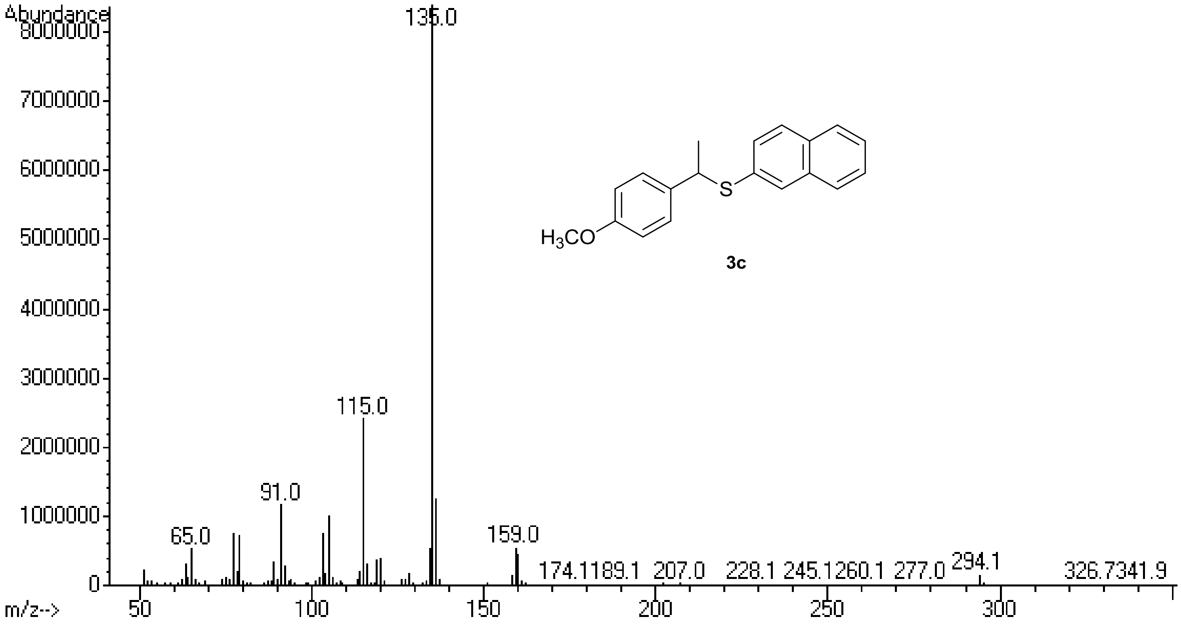
Abundance
8000000
7000000
6000000
5000000
4000000
3000000
2000000
1000000
0
m/z-->
50
100
150
200
250
300
135.0
115.0
91.0
65.0
159.0
174.1189.1 207.0 228.1 245.1260.1 277.0 294.1 326.7341.9
H3CO
S

3c

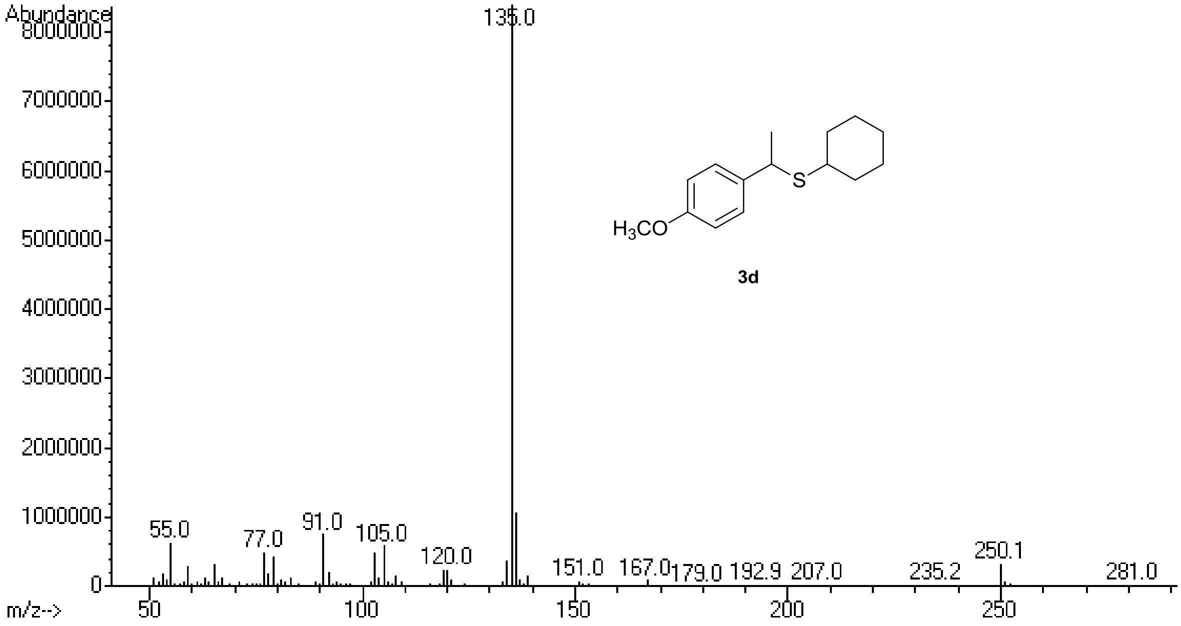
Abundance
8000000
7000000
6000000
5000000
4000000
3000000
2000000
1000000
0
m/z-->
50
100
150
200
250
135.0
55.0
77.0
91.0
105.0
120.0
151.0 167.0 179.0 192.9 207.0 235.2 250.1 281.0
H3CO
S

3d

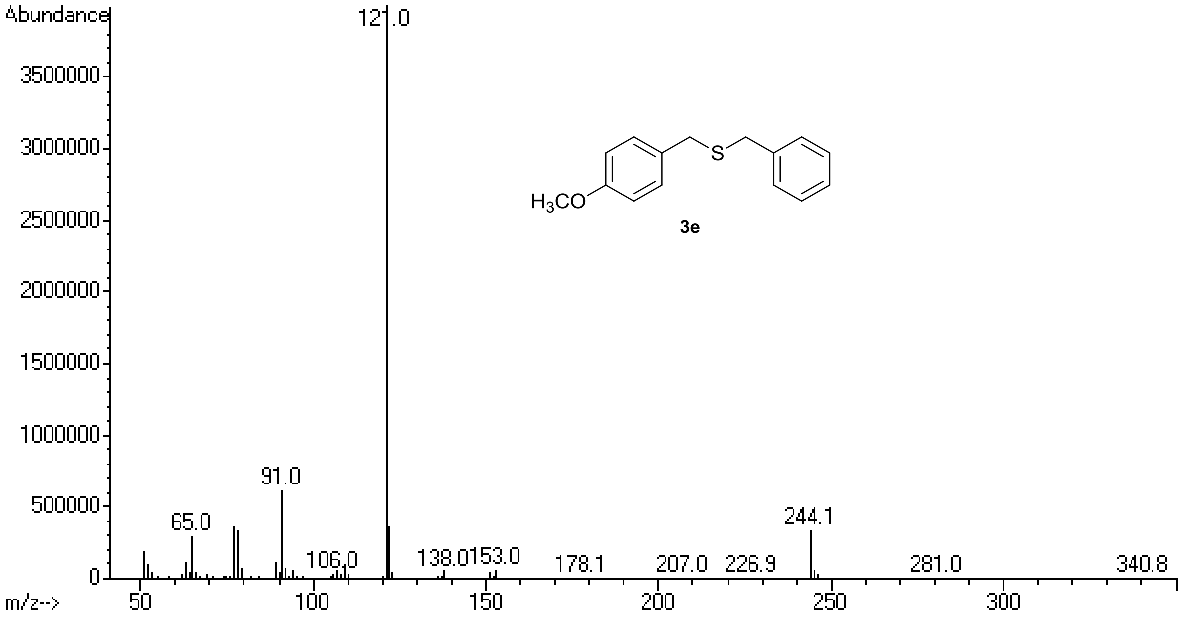
Abundance
m/z-->
121.0
91.0
65.0
106.0
138.0
153.0
178.1
207.0
226.9
244.1
281.0
340.8
H3CO
S
3e

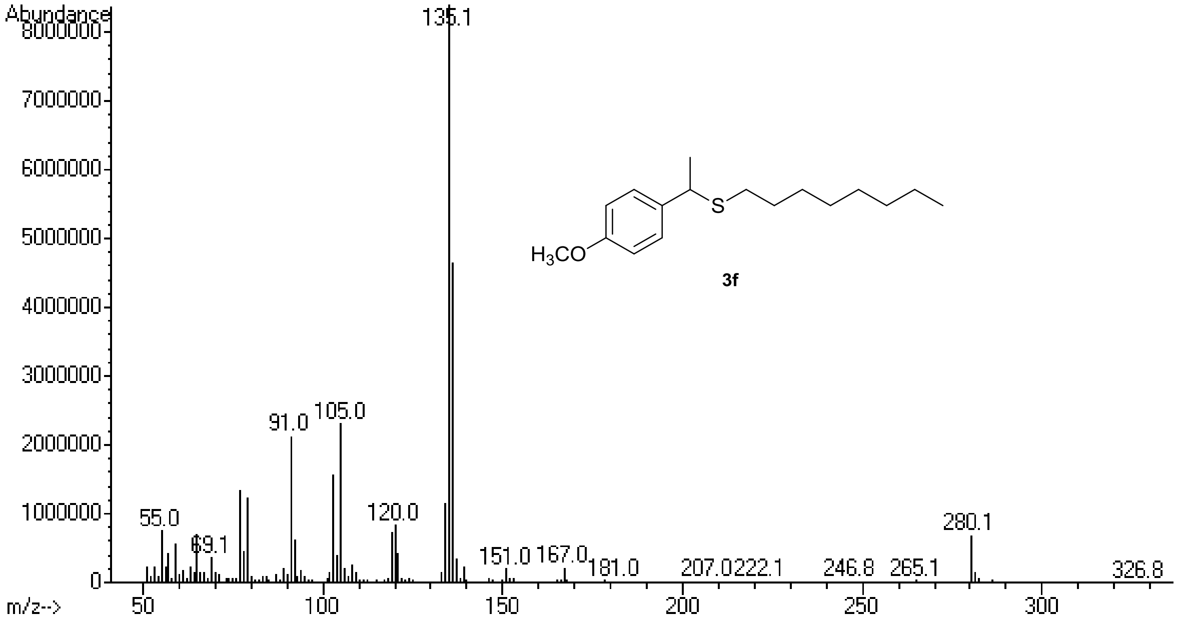
Abundance
m/z-->
135.1
91.0
105.0
55.0
69.1
120.0
151.0
167.0
181.0
207.0
222.1
246.8
265.1
280.1
326.8
H3CO
S
3f

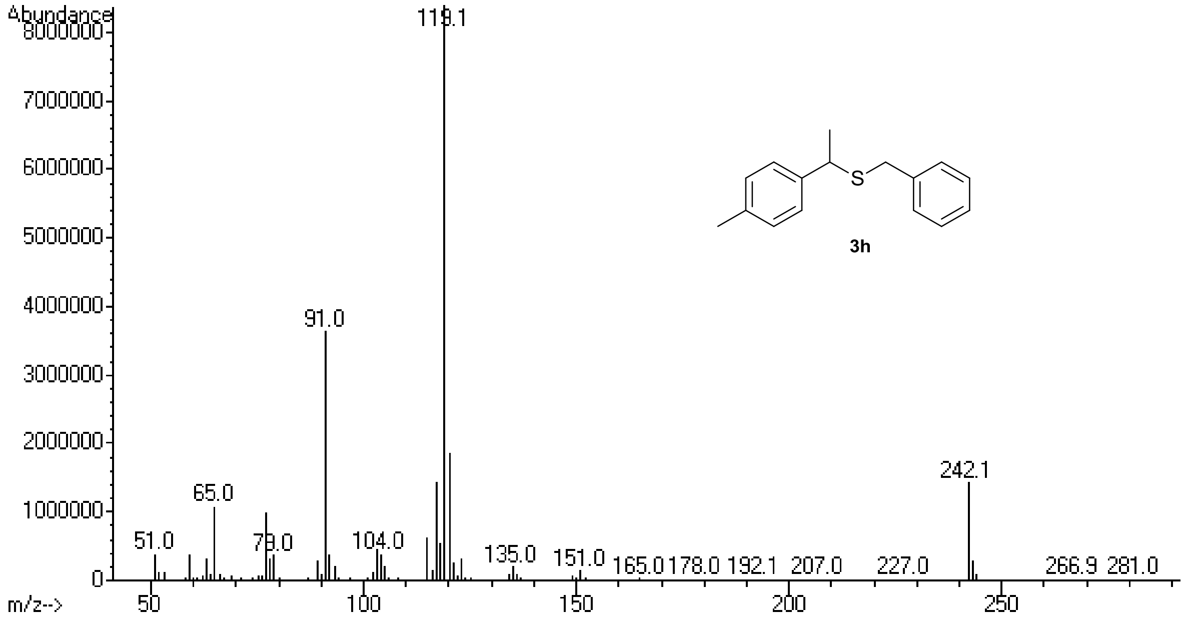
Abundance
8000000
7000000
6000000
5000000
4000000
3000000
2000000
1000000
0
m/z-->
50
100
150
200
250
119.1
91.0
65.0
51.0
79.0
104.0
135.0
151.0
165.0
178.0
192.1
207.0
227.0
242.1
266.9
281.0
S
3h

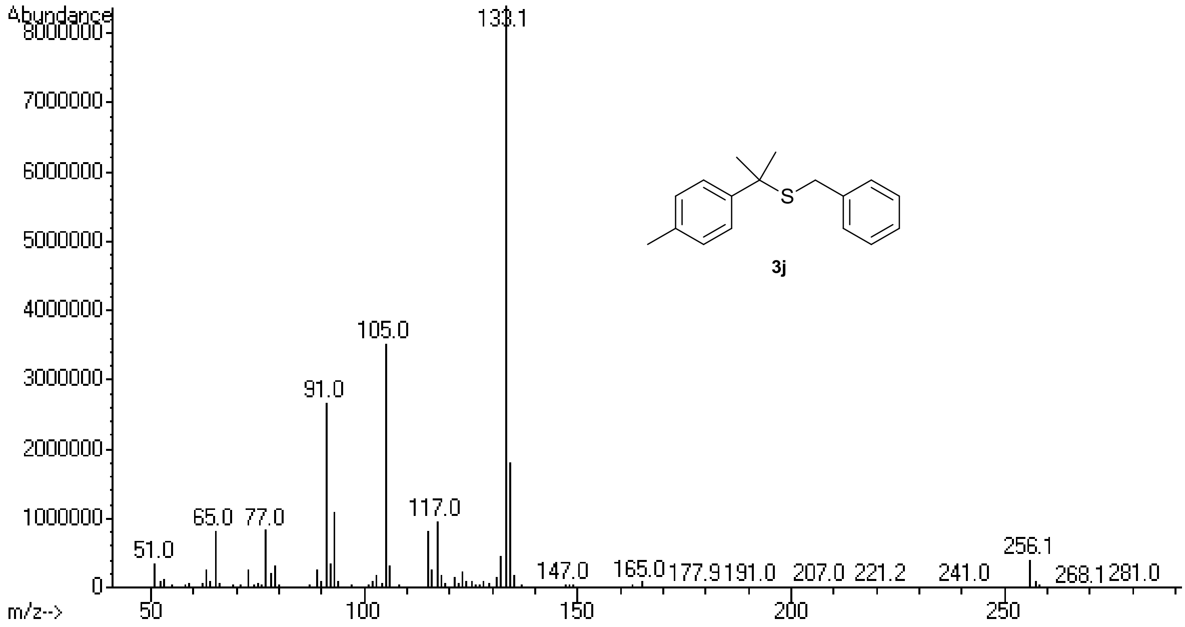
Abundance
8000000
7000000
6000000
5000000
4000000
3000000
2000000
1000000
0
m/z-->
50
100
150
200
250
133.1
105.0
91.0
117.0
65.0
77.0
51.0
147.0
165.0
177.9
191.0
207.0
221.2
241.0
256.1
268.1
281.0
S
3j

Optical Rotatory Power Determination

Optical rotatory power of (*R*)-1-phenylethanol and benzyl(1-phenylethyl)sulfane (**3q)** ethanol solutions were measured on a Jasco P-2000 digital polarimeter operating at the sodium D line with a 100 mm path length cell.

(*R*)-1-phenylethanol measured $[\alpha]_D^{21°C}$ (*c* = 0.05, EtOH) = +45.6).

benzyl(1-phenylethyl)sulfane (**3q)** measured $[\alpha]_D^{21°C}$ (*c* = 0.004, EtOH) = −0.65).
